# Supplementary material for: Predictors of time to death among under-five children in pastoral regions of Ethiopia: A retrospective follow-up study
Source: PLoS One. 2024 Jul 31;19(7):e0304662. doi: 10.1371/journal.pone.0304662 (PMC11290679; doi:10.1371/journal.pone.0304662)
Supplement: S1 Table — (DOCX) [file pone.0304662.s001.docx]

| **Additional file** | |  |  |
| --- | --- | --- | --- |
|  |  |  |  |
| **Additional file 1: Table S1.** Survival experience comparison | | | |
| **Ns** | **Variables** | **chi^2^(df)** | **LRT p-value** |
| 1 | Type of birth | 0.07 | 0.798 |
| 2 | Father's education level | 3.1 | 0.213 |
| 3 | Mother’s occupation | 49.09 | 0.001* |
| 4 | Region | 0.52 | 0.771 |
| 5 | Residence | 12.92 | 0.0003* |
| 6 | Toilet facilities | 0.67 | 0.412 |
| 7 | Mother’s education level | 0.13 | 0.717 |
| 8 | Sex of household head | 22.47 | 0.001* |
| 9 | Sex of child | 0.68 | 0.410 |
| 10 | ANC fellow up | 3.42 | 0.064 |
| 11 | Modern contraceptive used | 0.001 | 0.990 |
| 12 | Anemic Child | 25.08 | 0.001* |
| 13 | Exclusive breastfeeding | 20.36 | 0.001* |
| 14 | Mother's age | 22.25 | 0.001* |
| 15 | Child vaccinated | 37.47 | 0.001* |
| **LRT: Log-Rank Test.** | | | |
