# Supplementary material for: Predictors of time to death among under-five children in pastoral regions of Ethiopia: A retrospective follow-up study
Source: PLoS One. 2024 Jul 31;19(7):e0304662. doi: 10.1371/journal.pone.0304662 (PMC11290679; doi:10.1371/journal.pone.0304662)
Supplement: S1 Appendix — (DOCX) [file pone.0304662.s002.docx]

**Appendix**

STATA-codes for data Analysis

** This used to test Cox proportional assumption**

stset survival_time, failure(status==1) scale(1)

estat phtest, detail

** After the PH assumption had been checked, Analysis Bi-variable Cox PH model **

stset survival_time, failure(status==1) scale(1)

stcox i.Type_of_birth i.Fathers_educational_level i.Mothers_occupation i.Region i.Residence i.Toilet_facilities i.Mothers_educational_level i.Sex_of_household_head i.Sex_of_child i.ANC_fellow_up i.Modern_contraceptive_used i.Anemic_level_of_mothers i.Breastfeeding i.Mothers_age i.Child_vaccination_status

** After the Bi-variable Cox PH model analysis fit p-value <0.25 in Multivariable analysis **

**This used to model Multivariable Cox PH analysis**

stset survival_time, failure(status==1) scale(1)

stcox i.Mothers_occupation i.Residence i.Sex_of_household_head i.ANC_fellow_up i.Anemic_level_of_mothers i.Breastfeeding i.Mothers_age i.Child_vaccination_status

**This used for figures of KM-curves of category predictors**

stset survival_time, failure(status)

sts graph, by(i.Mothers_age) ytitle(Survival probability of under-five children) xtitle(Survival time of under-five children) title(KM-curve for Mother’s age differentials) subtitle(Fig 2: Survival curve by Mother’s age) legend(on)

stset survival_time, failure(status)

sts graph, by(i.Residence) ytitle(Survival probability of under-five children) xtitle(Survival time of under-five children) title(KM-curve for Residence place differentials) subtitle(Fig 2: Survival curve by residence place) legend(on)

stset survival_time, failure(status)

sts graph, by(i.Sex_of_household_head) ytitle(Survival probability of under-five children) xtitle(Survival time of under-five children) title(KM-curve for sex of household head differentials) subtitle(Fig 3: Survival curve by sex of household head) legend(on)

stset survival_time, failure(status)

sts graph, by(i.Anemic_level_of_mothers) ytitle(Survival probability of under-five children) xtitle(Survival time of under-five children) title(KM-curve for Anemic_level_of_mothers differentials) subtitle(Fig 4: Survival curve by Anemic_level_of_mothers) legend(on)

stset survival_time, failure(status)

sts graph, by(i.Child_vaccination_status) ytitle(Survival probability of under-five children) xtitle(Survival time of under-five children) title(KM-curve for Child vaccination status differentials) subtitle(Fig 5: Survival curve by Child vaccination status) legend(on)

stset survival_time, failure(status)

sts graph, by(i.Breastfeeding) ytitle(Survival probability of under-five children) xtitle(Survival time of under-five children) title(KM-curve for child breastfeeding differentials) subtitle(Fig 6: Survival curve by child breastfeeding) legend(on)

*The log-rank test was used to test whether there is significant d/ce b/n predictors.*

stset survival_time, failure(status==1) scale(1)

sts test Type_of_birth, logrank

sts test Fathers_educational_level, logrank

sts test Mothers_occupation, logrank

sts test Region, logrank

sts test Residence, logrank

sts test Toilet_facilities, logrank

sts test Mothers_educational_level, logrank

sts test Sex_of_household_head, logrank

sts test Sex_of_child, logrank

sts test ANC_fellow_up, logrank

sts test Modern_contraceptive_used, logrank

sts test Anemic_level_of_mothers, logrank

sts test Breastfeeding, logrank

sts test Mothers_age, logrank

sts test Child_vaccination_status, logrank
