# Supplementary material for: Predictors of time to death among under-five children in pastoral regions of Ethiopia: A retrospective follow-up study
Source: PLoS One. 2024 Jul 31;19(7):e0304662. doi: 10.1371/journal.pone.0304662 (PMC11290679; doi:10.1371/journal.pone.0304662)
Supplement: Attachment — Submitted filename: Manuscript (2).pdf [file pone.0304662.s005.pdf]

Submission date: 24-Jun-2023 10:44PM (UTC+0300)
Submission ID: 2121948783
File name: GGG.pdf (1.12M)
Word count: 9538
Character count: 49757

PLOS ONE

Time to death predictors among under-five children in pastoral regions of Ethiopia: a cross-sectional study

--Manuscript Draft--

**Manuscript Number:**	PONE-D-23-08757
**Article Type:**	Research Article
**Full Title:**	Time to death predictors among under-five children in pastoral regions of Ethiopia: a cross-sectional study
**Short Title:**	Time to death predictors among under-five children in pastoral regions of Ethiopia
**Corresponding Author:**	Bsrat Tesfay Hagos<br>Mekelle University College of Natural and Computational Sciences<br>Mekelle, ETHIOPIA
**Keywords:**	Time-to-death, Under-five children mortality, Cox PH model, Pastoral regions
**Abstract:**	Abstract<br>Background: Globally, although under-five children mortality rate substantially reduced over time; but, Sub-Saharan African countries including Ethiopia still continue to share the huge burden of under-five mortality. Ethiopia indicated a substantial reduction in under-five children mortality over time; however, the rate of decrease has varied across regional states. Therefore, the purpose of this study was to determine the major predictors, which contributed to shortened survival time-to-death of under-five children in pastoral regions of Ethiopia.<br>Methods: An institutionally-based retrospective cross-sectional study was conducted on under-five children in pastoral regions of Ethiopia from January 18, 2016 – June 27, 2016.<br>The Kaplan-Meier survival curve was used to estimate survival time, and the log-rank test was used to show the significant difference between categorical predictors. Both bivariable and multivariable Cox Proportional-Hazards (PH) model analyses were fitted to identify time-to-death predictor factors of under-five children.<br>Results: A total sample selected of 7,677 was included. The overall prevalence of under-five mortality was 84 per 1,000 live births . In the multivariable Cox PH model analysis, children vaccinated (AHR: 0.72, 95% CI: 0.59, 0.88), advanced mothers aged 35 – 40 (AHR = 1.27; 95% CI: 1.06, 1.52) and above 41 (AHR = 2.18; 95% CI:1.59, 2.98), not initiating exclusively breastfeeding (AHR: 1.26, 95% CI: 1.02, 1.55), the agriculture sector of mother's occupation (AHR: 2.57, 95% CI: 1.74, 3.81), male sex of household head (AHR: 0.67, 95% CI: 0.56, 0.81), not anemic Child (AHR: 0.67, 95% CI: 0.55, 0.83), and rural residence (AHR: 3.27, 95% CI: 1.45, 7.38) were found as significant predictors of time-to-death under-five children.<br>Conclusion: In this study, we found a higher rate of prevalence under-five mortality than the national figure. A child vaccinated, exclusively breastfeeding, mother's occupation, sex of household head, anemic Child, mother's age, and residence were found as the most influential predictor for time-to-death. Therefore, the government should give emphasis on the pastoral regional states to reduce the high prevalence of under-five mortality.
**Order of Authors:**	Bsrat Tesfay Hagos
	Gebru Gebremeskel Gebrerufael
**Additional Information:**	
**Question**	**Response**
**Financial Disclosure**<br><br>Enter a financial disclosure statement that describes the sources of funding for the work included in this submission. Review the submission guidelines for detailed	The funders had no role in study design, data collection and analysis, decision to publish, or preparation of the manuscript.

Powered by Editorial Manager® and ProduXion Manager® from Aries Systems Corporation

requirements. View published research articles from *PLOS ONE* for specific examples.<br><br>This statement is required for submission and **will appear in the published article** if the submission is accepted. Please make sure it is accurate.<br><br>**Unfunded studies**<br>Enter: *The author(s) received no specific funding for this work.*<br><br>**Funded studies**<br>Enter a statement with the following details:<br>• Initials of the authors who received each award<br>• Grant numbers awarded to each author<br>• The full name of each funder<br>• URL of each funder website<br>• Did the sponsors or funders play any role in the study design, data collection and analysis, decision to publish, or preparation of the manuscript?<br>• **NO** - Include this sentence at the end of your statement: *The funders had no role in study design, data collection and analysis, decision to publish, or preparation of the manuscript.*<br>• **YES** - Specify the role(s) played.<br><br>* typeset	
**Competing Interests**<br><br>Use the instructions below to enter a competing interest statement for this submission. On behalf of all authors, disclose any competing interests that could be perceived to bias this work—acknowledging all financial support and any other relevant financial or non-financial competing interests.<br><br>This statement is required for submission and **will appear in the published article** if the submission is accepted. Please make sure it is accurate and that any funding sources listed in your Funding Information later in the submission form are also declared in your Financial Disclosure	The authors have declared that no competing interests exist.

Powered by Editorial Manager® and ProduXion Manager® from Aries Systems Corporation

statement.<br><br>View published research articles from *PLOS ONE* for specific examples.<br><br>**NO authors have competing interests**<br><br>Enter: *The authors have declared that no competing interests exist.*<br><br>**Authors with competing interests**<br><br>Enter competing interest details beginning with this statement:<br><br>*I have read the journal's policy and the authors of this manuscript have the following competing interests: [insert competing interests here]*<br><br>* typeset	
**Ethics Statement**<br><br>Enter an ethics statement for this submission. This statement is required if the study involved:<br><br>• Human participants<br>• Human specimens or tissue<br>• Vertebrate animals or cephalopods<br>• Vertebrate embryos or tissues<br>• Field research<br><br>Write "N/A" if the submission does not require an ethics statement.<br><br>General guidance is provided below. Consult the submission guidelines for detailed instructions. **Make sure that all information entered here is included in the Methods section of the manuscript.**	A written approval letter was found to utilize the 2016 EDHS dataset from the DHS program for the present study. The IRB also approved the dataset for public utilize without personal, household identification. Therefore, the privacy of the participants was kept anonymous. Furthermore, per the DHS program discipline, and rule and regulations, this dataset was used for the present study only. Although, the dataset underlying the study can be accessed after legal registration at https://dhsprogram.com/data/available-datasets.cfm and writing a convincing letter about the descriptive objective of the project for the DHS program.

Powered by Editorial Manager® and ProduXion Manager® from Aries Systems Corporation

**Format for specific study types**<br><br>**Human Subject Research (involving human participants and/or tissue)**<br>• Give the name of the institutional review board or ethics committee that approved the study<br>• Include the approval number and/or a statement indicating approval of this research<br>• Indicate the form of consent obtained (written/oral) or the reason that consent was not obtained (e.g. the data were analyzed anonymously)<br><br>**Animal Research (involving vertebrate animals, embryos or tissues)**<br>• Provide the name of the Institutional Animal Care and Use Committee (IACUC) or other relevant ethics board that reviewed the study protocol, and indicate whether they approved this research or granted a formal waiver of ethical approval<br>• Include an approval number if one was obtained<br>• If the study involved *non-human primates*, add *additional details* about animal welfare and steps taken to ameliorate suffering<br>• If anesthesia, euthanasia, or any kind of animal sacrifice is part of the study, include briefly which substances and/or methods were applied<br><br>**Field Research**<br><br>Include the following details if this study involves the collection of plant, animal, or other materials from a natural setting:<br>• Field permit number<br>• Name of the institution or relevant body that granted permission	
**Data Availability**<br><br>Authors are required to make all data underlying the findings described fully available, without restriction, and from the time of publication. PLOS allows rare exceptions to address legal and ethical concerns. See the PLOS Data Policy and FAQ for detailed information.	No - some restrictions will apply

Powered by Editorial Manager® and ProduXion Manager® from Aries Systems Corporation

A Data Availability Statement describing where the data can be found is required at submission. Your answers to this question constitute the Data Availability Statement and **will be published in the article**, if accepted.<br><br>**Important:** Stating 'data available on request from the author' is not sufficient. If your data are only available upon request, select 'No' for the first question and explain your exceptional situation in the text box.<br><br>Do the authors confirm that all data underlying the findings described in their manuscript are fully available without restriction?	
**Describe where the data may be found in full sentences. If you are copying our sample text, replace any instances of XXX with the appropriate details.**<br><br>• If the data are **held or will be held in a public repository**, include URLs, accession numbers or DOIs. If this information will only be available after acceptance, indicate this by ticking the box below. For example: *All XXX files are available from the XXX database (accession number(s) XXX, XXX.)*.<br>• If the data are all contained **within the manuscript and/or Supporting Information files**, enter the following: *All relevant data are within the manuscript and its Supporting Information files.*<br>• If neither of these applies but you are able to provide **details of access elsewhere**, with or without limitations, please do so. For example:<br><br>*Data cannot be shared publicly because of [XXX]. Data are available from the XXX Institutional Data Access / Ethics Committee (contact via XXX) for researchers who meet the criteria for access to confidential data.*<br><br>*The data underlying the results presented in the study are available from (include the name of the third party*	The data set is available online and anyone can access it from www.measuredhs.com.

and contact information or URL).<br>• This text is appropriate if the data are owned by a third party and authors do not have permission to share the data.<br><br>* typeset	
Additional data availability information:	

Powered by Editorial Manager® and ProduXion Manager® from Aries Systems Corporation

Manuscript

Click here to access/download;Manuscript;Time to death predictors children-2016_6.docx

Time to death predictors among under-five children in pastoral regions of Ethiopia: a cross-sectional study

Gebru Gebremeskel Gebrerufael[1] and Bsrat Tesfay Hagos[2]

[1]Department of Statistics, College of Natural Science, Adigrat University, Adigrat, Ethiopia

[2]Department of Statistics, College of Natural Science, Mekelle University, Mekelle, Ethiopia

Email addresses:

GGG: gebrugebremeskel12@gmail.com

ORCID: https://orcid.org/0000-0003-2795-8529

BTH: bsrattesfaygu@gmail.com(Corresponding author)

Abstract

Background: *Globally, although under-five children mortality rate substantially reduced over time; but, Sub-Saharan African countries including Ethiopia still continue to share the huge burden of under-five mortality. Ethiopia indicated a substantial reduction in under-five children mortality over time; however, the rate of decrease has varied across regional states. Therefore, the purpose of this study was to determine the major predictors, which contributed to shortened survival time-to-death of under-five children in pastoral regions of Ethiopia.*

Methods: *An institutionally-based retrospective cross-sectional study was conducted on under-five children in pastoral regions of Ethiopia from January 18, 2016 – June 27, 2016. The Kaplan-Meier survival curve was used to estimate survival time, and the log-rank test was used to show the significant difference between categorical predictors. Both bivariable and multivariable Cox Proportional-Hazards (PH) model analyses were fitted to identify time-to-death predictor factors of under-five children.*

Results: *A total sample selected of 7,677 was included. The overall prevalence of under-five mortality was 84 per 1,000 live births. In the multivariable Cox PH model analysis, children vaccinated (AHR: 0.72, 95% CI: 0.59, 0.88), advanced mothers aged 35–40 (AHR = 1.27; 95% CI: 1.06, 1.52) and above 41 (AHR = 2.18; 95% CI:1.59, 2.98), not initiating exclusively breastfeeding (AHR: 1.26, 95% CI: 1.02, 1.55), the agriculture sector of mother's occupation (AHR: 2.57, 95% CI: 1.74, 3.31), male sex of household head (AHR: 0.67, 95% CI: 0.56, 0.81), not anemic Child (AHR: 0.67, 95% CI: 0.55, 0.83), and rural residence (AHR: 3.27, 95% CI: 1.45, 7.38) were found as significant predictors of time-to-death under-five children.*

Conclusion: *In this study, we found a higher rate of prevalence under-five mortality than the national figure. A child vaccinated, exclusively breastfeeding, mother's occupation, sex of household head, anemic Child, mother's age, and residence were found as the most influential*

predictor for time-to-death. Therefore, the government should give emphasis on the pastoral regional states to reduce the high prevalence of under-five mortality.

Keywords: *Time-to-death, Under-five children mortality, Cox PH model, Pastoral regions*

Background

The under-5 children mortality rate (U5CMR) is defined as the probability of a child born in a specified year dying between birth and the fifth birthday [1]. The 1st five years of life are the most important years for the intellectual and physical development of children [1].

Globally, although there is a reduction in U5CMR, from 5.9 million deaths of children in 2015 to 5.3 million in 2018; however, there still remains a high mortality rate in African countries nearly one-twelfth (81 deaths per 1,000 live births) including Ethiopia, which is about seven times higher than in the European countries [1, 2].

In Africa, more than 25% of all death rates occur among under–five years of children worldwide [3]. Among the African countries Somalia (133/1000 live births), Chad (127/1000 live births), Central African Republic (124/1000 live births), Sierra Leone (114/1000 live births), Mali (111/1000 live births), and Nigeria (104/1000 live births) were the top 6 countries with U5CMR accounting for more than 50% under-five children death rate in Sub–Saharan African (SSA) countries and about one-fifth of under-five mortality worldwide in 2016 [3].

The SSA continues to be the region with the highest U5CMR in the world with one child in every eleven live birth dying before 5 years of age [4, 5]. Therefore, under-five children mortality is still a serious SSA public health concern [5]. Similarly, U5CMR in Ethiopia is one of the most and biggest difficult public health problems that should be given emphasis [6].

The fourth Millennium Development Goal (MDG4) was aimed at decreasing under-five children mortality by two–thirds (67%) and this was unfinished agenda for most of the low and middle-income countries [7].

With the present death rates of children in Ethiopia achieving the sustainable development goal, which is decreasing U5CMR to 55 deaths per 1,000 live births by 2030 is challenging and investigations indicated that there is an important geographical regional variation in under-five mortality [6]. This means that U5CMR in Ethiopia is still higher (i.e., reported to be 67 deaths per 1,000 live births) and the largest figure was recorded in Somali, Benishangul–Gumuz, and Afar national regional states where there was estimated to be about 94, 98, and 125 deaths per 1,000 live birth, respectively [8]. Besides, evidence from different literature displayed that an anemic Child, low mother's education level, not initiating breastfeeding, Ante–Natal Care

(ANC) fellow up, advanced mother's age, and not vaccinated Child is among the predictor factors associated with U5CMR [1, 3, 9–11].

To alleviate these problems, Ethiopia is one of the SSA countries working on the reduction of U5CMR, through improving coverage, quality, and use of skilled care, important newborn care and management of preterm and low birth weight with other different interventions have been made by the government. Nonetheless, it still remained very high in the pastoral regions of Ethiopia (i.e., reported to be in Somalia, Benishangul-Gumuz, and Afar) [7, 8].

Significance of the study

Results obtained from this present study will inform planning interventions to improve the survival time of under-five children in the study area and similar areas of Ethiopia. Moreover, this present study will serve as baseline information for additional studies.

Objective of the study

General objective

The general purpose of this study was to determine the major predictors, which contributed to shortened survival time-to-death of under-five children in pastoral regions of Ethiopia.

Specific objectives

➢To determine the prevalence of mortality among under-five children in the pastoral regions of Ethiopia

➢To identify predictors associated with time-to-death among under-five children in the pastoral regions of Ethiopia

➢To estimate the survival time and compare survival curves between the different categories of predictors associated with time-to-death among under-five children.

Methods

Study design, source of data, and period

This study has used an institutionally-based retrospective study and secondary data analysis based on the 2016 Ethiopia Demographic and Health Survey (EDHS). The 2016 EDHS was conducted under the support of the Ministry of Health (MOH) and was implemented by the Central Statistical Agency (CSA) and partner organizations from January 18, 2016 – June 27, 2016, with a nationally representative sample selected of nearly 16,650 households. All women of reproductive age (15–49) and all men age of the group (15–59) in these households were eligible for individual interviews.

Sampling technique, inclusion and exclusion criteria

The EDHS implemented a two-stage stratified sample design. The country has nine regional states and two administrative cities. In the first stage, 645 enumeration areas (443 in rural areas and 202 in urban areas) were selected with probability proportional to size. In the second stage, 28 households were involved with a cluster of equal probability systematic selection from the newly created household list. Afar, Benishangul-Gumuz, and Somali regional states were selected since based on the 2016 EDHS report, these regional states had the highest U5CMR. Among these regions, a total sample selected of 7,677 women with a live birth of (642 events and 7,035 censored children) in the 5 years preceding the survey were included for this study analysis. The detailed diagrammatic sample selection procedure was presented (see **Figure. 1**).

Study variables

Response variable

The response variable for this study was considered to be the length (survival) of time measured in months from date of birth until time-to-death (censor) on under-five children. The outcomes of under-five children were dichotomized as death (event) and censored category outcomes which were measured in months. Censored referred as to under-five children who were alive at the end of follow-up. The purposes of this study, particularly the times for the under-five children who were died treated as event times and other outcomes assumed as right-censored.

Predictor variables

Time-to-death predictors among under-five children in this study were selected from the available similar studies on the subject; the main predictors associated with time-to-death among under-five children are presented in Table 1.

Table 1: Operational definition and categorization of predictor variables used in the study

S.N	Variables	Description and categorization
1	Type of birth	Type of birth child's (single, multiple)
2	Father's education level	Father's education level (no education, primary, secondary and above)
3	Mother's occupation	Mother's occupational status (not working, non-agriculture sector, agriculture sector)
4	Region	Region (Affar, Somalia, Benshangul–gumuaz)
5	Residence	Residence place (urban, rural)
6	Toilet facilities	Toilet facilities of mother's (no facility, with facility)
7	Mother's education level	Mother's education level (no education, primary and above)
8	Sex of household head	Sex of household head (female, male)

9	Sex of child	Sex of child (male, female)
10	ANC fellow-up	Ante-Natal care (ANC) fellow up (no, yes)
11	Modern contraceptive used	Modern contraceptive method used (no, yes)
12	Anemic Child	Anemic level of child's (yes, no)
13	Exclusively breastfeeding	Exclusively breastfeeding (yes, no)
14	Mother's age	Mother's age (in year) (34 and below, 35–40, 41 and above)
15	Child vaccinated	Child vaccinated status (no, yes)

Statistical methods of data analysis

In the bivariable STATA software version 14 package (i.e., the data was extracting, recording, and analysis). The study was done descriptive statistics such as frequency distributions and percentages to explore the entire variables of the study. The Kaplan-Meier (K-M) estimators were applied to show the survival experience of children over time for categorical predictor variables. The log-rank test was used to (compare the survival curves) test whether there is a significant difference between predictor variables towards the survival ability of under-five children (see Additional file 1: Table S1).

One of the most common types of regression models used in survival analysis is the Cox Proportional-Hazard (PH) model. Cox Proportional-Hazard (PH) regression model, $h(t) = h_0(t) * exp^{(b_1x_1 + b_2x_2 + b_3x_3 + \ldots\ldots\ldots\ldots b_px_p)}$ were used to identify and to check the impact of each independent variables on the time-to-death. The hazard function $h(t)$ is determined by a set of p covariates ($x_1, x_2, x_3, \ldots\ldots, x_p$), whose impact is measured by the size of the respective coefficients ($b_1, b_2, b_3, \ldots\ldots, b_p$). The term h_0(t) is called the baseline hazard for a time-to-death.

The proportionality assumptions of Cox PH regression model analysis were assured on all each predictor variables and on the global test of proportionality (Shoenfield residual test). After the proportional hazard assumption had been checked, the bivariable Cox PH regression model was fitted for each predictor variable. Furthermore, those variables having p-value ≤ 0.25 in the bivariable model analysis were included in the multivariable Cox PH regression model. Crude and adjusted hazard ratios with a 95% Confidence Interval (CI) were used to measure the strength of association and to explore statistically significant predictors. In the multivariable Cox PH regression model analysis, variables having P-value < 0.05 were found as significant predictors of time-to-death on under-five children. The goodness of fit of the final model was checked by the Likelihood Ratio Test (LRT).

Operational definitions

Survival time: In this paper, survival time referred to the period from starting of observation until the occurrence of the outcome of observation (event or censored).

Event: In this study, the event referred to the study subject who had experienced the interest of occurrence outcome (had died) during the observation period and labeled as (1).

Censored: Censored is referred to the study of under-five children who were alive at the end of follow-up.

Ethical consideration

A written approval letter was found to utilize the 2016 EDHS dataset from the DHS program for the present study. The IRB also approved the dataset for public utilize without personal, household identification. Therefore, the privacy of the participants was kept anonymous. Furthermore, per the DHS program discipline, and rule and regulations, this dataset was used for the present study only. Although, the dataset underlying the study can be accessed after legal registration at https://dhsprogram.com/data/available-datasets.cfm and writing a convincing letter about the descriptive objective of the project for the DHS program.

Results

Descriptive statistics

In this study, a total sample selected of 7,677 was included in the analysis, with 642 under-five deaths. The majority (96.6%) and (84.7%) of the study participants lived in rural resident and had no education level, respectively. Approximately, 98.6% of study participants were from single birth type and 49.2% were from the Somali regional state. About 79.1% of respondents were having toilet facilities and the majorities (64.2%) of participants were from the female sex of household heads. Concerning the use of the modern contraceptive method, 94.1% of respondents were not used the modern contraceptive method.

Most (75.6%) fathers have no education level and 64.3% of mothers did not have ANC follow-up during their last pregnancy. Moreover, 51.9% of children were males and the majorities (79.1%) of children were not anemic (Table 2).

Table 2: Mother's and child's socio-demographic characteristics, health and environmental predictors in the pastoral regions of Ethiopia, 2016 (N = 7,677)

Variables	Frequency (N)	Percentage (%)
Type of birth		
Single	7572	98.6
Multiple	105	1.4

Father's education level		
No education	5803	75.6
Primary	1086	14.1
Secondary and above	788	10.3
Mother’s occupation		
Not working	5722	74.5
Non-agriculture sector	1680	21.9
Agriculture sector	275	3.6
Region		
Affar	2287	29.8
Somalia	3775	49.2
Benshangul-gumuaz	1615	21
Residence		
Urban	261	3.4
Rural	7416	96.6
Toilet facilities		
No facility	1606	20.9
With facility	6071	79.1
Mother’s education level		
No education	6502	84.7
Primary and above	1175	15.3
Sex of household head		
Female	4931	64.2
Male	2746	35.8
Sex of child		
Male	3986	51.9
Female	3691	48.1
ANC fellow-up		
No	4937	64.3
Yes	2740	35.7
Modern contraceptive used		
No	7224	94.1

Yes	453	5.9
Anemic Child		
Yes	1605	20.9
No	6072	79.1
Exclusively breastfeeding		
Yes	4738	61.7
No	2939	38.3
Mother's age		
34 and below	3363	43.8
35–40	3780	49.2
41 and above	534	7
Child vaccinated		
No	3058	39.8
Yes	4619	60.2

Comparison of the survival ability of under-five children

Besides, the descriptive statistics given above in **Table 2**, Kaplan-Meier (K-M) survival curve estimators are plotted for the most essential predictor variables (See **Figures 2 – 7**). These samples of K-M curves show that under-five year's old children whose advanced mother's age, rural residence, being male sex household head, being anemic Child, not vaccinated Child, and not initiating exclusive breastfeeding had shorted survival time compared to those reference categories. Additionally, from the log-rank test, there was a significant difference between the mother's age (P-value = 0.000), residence groups (P-value = 0.000), sex of household head (P-value = 0.000), anemic Child (P-value = 0.000), vaccinated Child (P-value = 0.000), and exclusive breastfeeding (P-value = 0.000) on the time-to-death among under-five children (see Additional file 1: Table S1).

Time-to-death predictors among under-five children

In this study, the overall prevalence of U5CMR in the study period was 84 deaths per 1,000 live births. None of the predictors failed the Cox PH regression model required assumption (see **Table 3**).

Table 3: Test of proportional-hazards assumption (STATA software version 14)

Variables	Rho	Chi^2	P-value
Type of birth (reff.= single)			
Multiple	-0.024	0.3	0.5808
Father's education level (reff.= no education)			
Primary	0.0537	1.46	0.2275
Secondary and above	-0.024	0.29	0.5923
Mother's occupation (reff.= not working)			
Non-agriculture sector	0.07202	2.76	0.0964
Agriculture sector	-0.0201	0.23	0.6347
Region (reff.= Affar)			
Somalia	-0.01	0.06	0.8087
Benshangul-gumuaz	-0.028	0.48	0.4884
Residence (reff.= urban)			
Rural	-0.068	2.68	0.1018
Toilet facilities (reff.= no facility)			
With facility	0.029	0.55	0.4597
Mother's education level (reff.= no education)			
Primary and above	-0.047	1.23	0.2675
Sex of household head (reff.= female)			
Male	-0.006	0.02	0.8878
Sex of child (reff.= male)			
Female	-0.04	0.86	0.3527
ANC fellow up (reff.= no)			
Yes	0.0291	0.48	0.4876
Modern contraceptive used (reff.= no)			
Yes	-0.036	0.75	0.3874
Anemic Child (reff.= yes)			
No	-0.023	0.28	0.5944
Exclusively breastfeeding (reff.= yes)			
No	-0.033	0.62	0.4321
Mother's age (reff.= 34 and below)			

35–40	0.0744	3.02	0.08[illegible]
41 and above	0.04422	1.07	0.30[illegible]
Child vaccinated (reff.= no)			
Yes	0.03674	0.76	0.38[illegible]
Global test	-------	18.56	0.48[illegible]

N.B: Chisq refers to chi-square statistic value; Rho refers to the correlation among covariates and survival times.

In the bivariable Cox PH regression model analysis, significant predictors (p-value ≤ 0.25) of U5CMR included: type of birth, father's education level, region, mother's occupation, residence, toilet facilities, mother's education level, sex of household head, sex of the child, ANC fellow-up, modern contraceptive used, anemic Child, exclusively breastfeeding, mother's age, and Child vaccinated.

In the multivariable Cox PH regression model analysis, only eight variables were identified as significant predictors of time-to-death in under-five children. Accordingly, the critical risk factors of time-to-death among children who were born to mothers with advanced age were 1.27 and 2.18 times higher as compared to their reference (Adjusted Hazard Ratio (AHR) = 1.27; 95% CI: 1.06, 1.52) and (AHR = 2.18; 95% CI: 1.59, 2.98), respectively. Children delivered by agriculture sector mothers occupation were 2.57 (AHR: 2.57, 95% CI: 1.74, 3.31) times more likely to die as compared to not working mothers occupation.

For the variable sex of household head, the risks of under-five mortality were about 33% times (AHR: 0.67, 95% CI: 0.56, 0.81) lower for children from the male household head than the female counterpart. Children who had not been anemic were 33% times (AHR: 0.67, 95% CI: 0.55, 0.83) lower likely to die as compared to anemic children. In addition, children who did not initiate exclusively breastfeeding were 1.26 times (AHR: 1.26, 95% CI: 1.02, 1.55) more likely to die as compared to exclusively breastfeeding children. Furthermore, Child vaccination was found significantly associated with time to death in under-five children. The risks of under-five time-to-death were about 38% times (AHR: 0.72, 95% CI: 0.59, 0.88) lower among children vaccinated than those not vaccinated children. Finally, the residence had a negative impact on time-to-death among under-five children. The risks of under-five children mortality were 3.27 times (AHR: 3.27, 95% CI: 1.45, 7.38) higher among children belonging to a mother who lived in rural than those who lived in urban areas (Table 4).

Cox PH regression model and model adequacy checking results

Cox PH regression model analysis was used to investigate the effect of each covariate on the time-to-death of under-five children. The Likelihood Ratio Test (LRT) of the Cox PH regression model analysis with the Breslow method was used to test model adequacy. The test statistic was 142.32 with a *p*-value of 0.000, which showed that the model is a good fit for the data set at a 5% level of significance (**Table 4**).

Table 4: Bivariable and multivariable Cox PH regression model analysis of time-to-death predictors among under-five children in the pastoral regions of Ethiopia, 2016 (N = 7,677)

	Survival status				
Variables	Censored	Event	CHR (95% CI)	AHR (95% CI)	P-value
Mother’s occupation					
Not working (reff.)	5263	459	1	1	
Non-agriculture sector	1547	133	0.64 (0.52, 0.8)*	0.78 (0.63, 0.98)*	0.036*
Agriculture sector	225	50	2.1 (1.54, 2.86)*	2.57 (1.74, 3.31)*	0.000*
Residence					
Urban (reff.)	250	11	1	1	
Rural	6785	631	3.88 (1.74, 8.69)*	3.27 (1.45, 7.38)*	0.004*
Sex of house hold head					
Female (reff.)	4494	437	1	1	
Male	2541	205	0.65 (0.54, 0.78)*	0.67 (0.56, 0.81)*	0.007*
ANC fellow up					
No (reff.)	4490	447	1	1	
Yes	2545	195	0.84 (0.70, 1.01)	0.89 (0.73, 1.08)*	0.231
Anemic Child					
Yes (reff.)	1452	153	1	1	
No	5583	489	0.61 (0.50, 0.74)*	0.67 (0.55, 0.83)*	0.000*
Exclusively breastfeeding					
Yes (reff.)	4310	428	1	1	
No	2725	214	1.52 (1.26, 1.83)*	1.26 (1.02, 1.55)*	0.030*
Mother's age					
34 and below	3100	263	1	1	
35–40	3459	321	1.29 (1.08, 1.55)*	1.27 (1.06, 1.52)*	0.010*
41 and above	476	58	2.0 (1.147, 2.74)*	2.18 (1.59, 2.98)*	0.000*

Child vaccinated (reff.= no)					
No (reff.)	2809	249	1	1	
Yes	4226	393	0.58 (0.48, 0.70)*	0.72 (0.59, 0.88)*	0.002*

NB: *Significant at p-value < 0.05.

Abbreviations: reff.: reference

$LRT = LR\ chi^2(10) = 142.32\ (p < 0.000)$

Discussion

In spite of numerous improvements and interventions made to recover the survival of newborns, U5CMR remains a major SSA public health concern; particularly in Ethiopia is largely affected. Therefore, we conducted this retrospective cross-sectional study to determine U5CMR in the pastoral regions of Ethiopia. Accordingly, the overall prevalence of U5CMR in the pastoral regions of Ethiopia was found to be 84 deaths per 1,000 live births. This finding is consistent with a previous study conducted in SSA (81 deaths per 1,000 live births) [3], Ethiopia (61 deaths per 1,000 live births) [1], and Nigeria (104 deaths per 1,000 live births). However, our finding is lower than the results of numerous studies conducted in Ethiopia (273 deaths per 1,000 live births), Somalia (133 deaths per 1,000 live births), Chad (127 deaths per 1,000 live births), Central African Republic (124 deaths per 1,000 live births), Sierra Leone (114 deaths per 1,000 live births), Mali (111 deaths per 1,000 live births) [3], and Ghana (250 deaths per 1,000 live births) [10].

Conversely, our finding is much higher than the U5CMR prevalence reported in northern Ethiopia (35.62 per 1,000 live births) [5], the Somali region of Ethiopia (57 deaths per 1,000 live births) [12], the rural area of Eastern Ethiopia (28.37 deaths per 1,000 live births) [13], Tigray region Northern Ethiopia (62.5 deaths per 1,000 live births) 62.5 [14], and Ethiopia (36.7 deaths per 1,000 live births) [15]. The above differences between studies might be explained, by the variations in study design, sample size, study period, study settings, and socio-demographic characteristics of study participants.

In this study, the risk rate of time–to–death among under-five children with not have anemic was 33% times lower than among children with anemic. A study conducted in Ethiopia, Jimma University Specialized Hospital, Gedeo Zone, and Sekota Waghemra Zone of northern Ethiopia also showed the case fatality rate of severe anemic was lower [9, 16–18]. Similarly, studies conducted in South Africa and Niger [19, 20] supported that the hazard of under-five children mortality is higher in exposed children. This is because, during an anemic, the prevalence of

infection and the probability of heart failure increase, and overall compliance will reduce. The other possible explanation of this study might be that anemic children can lead to growth retardation and prematurity, which are the main associated factors for under-five children mortality.

For the predictor variable sex of household heads, the risks of time-to-death under-five children were lower among male household heads than that female household heads. The risks of time-to-death on under-five children were the lowest among children from mothers who had less number of total children ever born [21–23].

Moreover, exclusive breastfeeding practice was a significant predictor of time-to-death among under-five children. Our study displayed that children who did not initiate breastfeeding exclusively were at a higher risk of death as compared to their exclusively breastfeed counterparts. This finding is consistent with a study conducted in Ethiopia [1]. A study conducted in other SSA countries showed that if breastfeeding was started within the first day of birth, the risk of time-to-death among under-five children reduced by 16% and could be reduced by 22% if it is initiated within one hour [24].

Mother's occupation was significantly associated with time-to-death among under-five children. Accordingly, children delivered by mothers who work in the agriculture sector were more likely to die as compared to their mothers not working. This finding contradicts findings from a study reported from Ethiopia, which shows the hazards of under-five mortality were lower among children born from not working mothers than mothers who work in the agriculture sector [21]. The possible explanation for these contradictory findings might be due to the difference in the socio-economic and demographic status of the mothers, as agriculture sector mothers have a better income than not working mothers.

In the current study, it was observed that the critical risk factors of time-to-death among under-five children born increases as to mother's age increases. This finding is consistent with other studies conducted in Sudan [25], and Suriname [26]. This could be connected to the evidence-based facts that older mothers' age is associated with certain pregnancy-connected risks including poor newborn outcomes [27]. However, this study was in contrast to another study in Nigeria [28]. Therefore, interventions need to be cooperated to reduce risky conditions such as pregnancy in older mothers' age. Another important socio-demographic factor significantly associated with time-to-death on under-five children with the residence. Rural residents' children were at a higher risk of time-to-death on under-five children compared to urban residents. This finding is consistent with a study conducted in Ethiopia [29–31]. Finally, this study showed that

children who were not vaccinated were at a higher risk of time-to-death as compared to those who were vaccinated. This finding is consistent with a study conducted in Ethiopia [11].

Strength and limitation of the study

This present study was based on a nationally representative data set with large sample size. Also, it was according to the most appropriate statistical Cox PH regression model analysis (survival model approach) to accommodate the public or regional level variability of under-five children mortality. Moreover, since it is according to the national survey data set the study has the potential to give understanding to policymakers and program designers to plan suitable intervention strategies both at the national and regional levels. However, this study had limitations in that the EDHS is mostly according to respondents' self-report and might have the possibility of recall bias since the interview was retrospective study design and the presence of high missing values on the 2016 EDHS data; some important predictor variables are not included in the study like gestational mothers age.

To complement the limitations of this study, the investigators should be recommended that robust parametric models were better rather than traditional Cox PH regression methods when their data structure is hierarchical as on the 2016 EDHS data set and further study using prospective study design would better compensate these limitations.

Conclusion

In this study, we found a higher rate of prevalence of under-five mortality than the national figure. Child vaccination, exclusively breastfeeding, mother's occupation, sex of household head, anemic Child, residence, and mother's age were found as critical risk factors significantly associated with time-to-death among under-five children. Based on our findings, we strongly recommend that special emphasis shall be given to under-five children mortality in the pastoral regions of Ethiopia. Therefore, public health interventions and improvements that increase maternal health service utilization such as Child vaccination, exclusive breastfeeding, and family planning service utilization to increase Child survival time are needed to lengthen the time-to-death of under-five children in these regions of Ethiopia.

Abbreviations

AHR: **A**djusted **H**azard **R**atio; ANC: **A**nte-**N**atal **C**are; CHR: **C**rude **H**azard **R**atio; CI: **C**onfidence **I**nterval; CSA: **C**entral **S**tatistical **A**gency; EDHS: **E**thiopia **D**emographic **H**ealth **S**urvey; KM: **K**aplan-**M**eier; LRT: **L**ikelihood **R**atio **T**est; MDG4: **F**ourth **M**illennium **D**evelopment **G**oal; MOH: **M**inister of **H**ealth; PH: **P**roportional-**H**azards; SSA: **S**ub-**S**aharan **A**frican; U5CMR: **U**nder-**5** **C**hildren **M**ortality **R**ate

Acknowledgments

We greatly acknowledge MEASURE DHS for permitting access to the 2016 EDHS data set.

Authors' contributions

BTH conceived the study. GGG and BTH analyzed the data, drafted the manuscript, and extensively reviewed the article. All authors have read and approved the final manuscript.

Funding

No funding was obtained for this study.

Availability of data and materials

The data set is available online and anyone can access it from www.measuredhs.com.

Declarations

The ethics approval and consent to participate since the study was a secondary data analysis of publicly available survey data from the MEASURE DHS program, ethical approval and participant consent were not necessary. We requested DHS Program and permission was granted to download and use the data from http://www.dhsprogram.com. There are no names of individual (household) addresses in the data files.

Consent for publication

Not applicable.

Competing interests

The authors declare that they have no conflict of interest.

Author details

[1]Department of Statistics, College of Natural Science, Adigrat University, Adigrat, Ethiopia

[2]Department of Statistics, College of Natural Science, Mekelle University, Mekelle, Ethiopia

Appendix

STATA-codes for data Analysis

```
** This used to test Cox proportional assumption**
stset survival_time, failure(status==1) scale(1)
estat phtest, detail

** After the PH assumption had been checked, Analysis Bi-variable Cox PH model **
stset survival_time, failure(status==1) scale(1)
stcox i.Type_of_birth i.Fathers_educational_level i.Mothers_occupation i.Region i.Residence i.Toilet_facilities i.Mothers_educational_level i.Sex_of_household_head i.Sex_of_child i.ANC_fellow_up i.Modern_contraceptive_used i.Anemic_level_of_mothers i.Breastfeeding i.Mothers_age i.Child_vaccination_status

** After the Bi-variable Cox PH model analysis fit p-value <0.25 in Multivariable analysis **
**This used to model Multivariable Cox PH analysis**
stset survival_time, failure(status==1) scale(1)
stcox i.Mothers_occupation i.Residence i.Sex_of_household_head i.ANC_fellow_up i.Anemic_level_of_mothers i.Breastfeeding i.Mothers_age i.Child_vaccination_status

**This used for figures of KM-curves of category predictors**
stset survival_time, failure(status)
sts graph, by(i.Mothers_age) ytitle(Survival probability of under-five children) xtitle(Survival time of under-five children) title(KM-curve for Mother's age differentials) subtitle(Fig 1: Survival curve by Mother's age) legend(on)

stset survival_time, failure(status)
sts graph, by(i.Residence) ytitle(Survival probability of under-five children) xtitle(Survival time of under-five children) title(KM-curve for Residence place differentials) subtitle(Fig 2: Survival curve by residence place) legend(on)

stset survival_time, failure(status)
sts graph, by(i.Sex_of_household_head) ytitle(Survival probability of under-five children) xtitle(Survival time of under-five children) title(KM-curve for sex of household head differentials) subtitle(Fig 3: Survival curve by sex of household head) legend(on)

stset survival_time, failure(status)
```

```
sts graph, by(i.Anemic_level_of_mothers) ytitle(Survival probability of under-five children) xtitle(Survival time of under-five children) title(KM-curve for Anemic_level_of_mothers differentials) subtitle(Fig 4: Survival curve by Anemic_level_of_mothers) legend(on)

stset survival_time, failure(status)
sts graph, by(i.Child_vaccination_status) ytitle(Survival probability of under-five children) xtitle(Survival time of under-five children) title(KM-curve for Child vaccination status differentials) subtitle(Fig 5: Survival curve by Child vaccination status) legend(on)

stset survival_time, failure(status)
sts graph, by(i.Breastfeeding) ytitle(Survival probability of under-five children) xtitle(Survival time of under-five children) title(KM-curve for child breastfeeding differentials) subtitle(Fig 6: Survival curve by child breastfeeding) legend(on)
*The log-rank test was used to test whether there is significant d/ce b/n predictors.*
stset survival_time, failure(status==1) scale(1)
sts test Type_of_birth, logrank
sts test Fathers_educational_level, logrank
sts test Mothers_occupation, logrank
sts test Region, logrank
sts test Residence, logrank
sts test Toilet_facilities, logrank
sts test Mothers_educational_level, logrank
sts test Sex_of_household_head, logrank
sts test Sex_of_child, logrank
sts test ANC_fellow_up, logrank
sts test Modern_contraceptive_used, logrank
sts test Anemic_level_of_mothers, logrank
sts test Breastfeeding, logrank
sts test Mothers_age, logrank
sts test Child_vaccination_status, logrank
```

Reference

1. Zewudie AT, Gelagay AA, Enyew EF. Determinants of under-five child mortality in Ethiopia: analysis using Ethiopian demographic health survey, 2016. Int J Pediatr. 2020; 2020. https://doi.org/10.1155/2020/7471545.
2. Gebretsadik S, Gabreyohannes E. Determinants of under-five mortality in high mortality regions of Ethiopia: an analysis of the 2011 Ethiopia demographic and health survey data. Int J Popul Res. 2016.
3. Woldeamanuel BT. Socioeconomic, Demographic, and Environmental Determinants of Under-5 Mortality in Ethiopia: Evidence from Ethiopian Demographic and Health Survey, 2016. Child Development Research. 2019.https://doi.org/10.1155/2019/1073782.
4. L. Alkema, D. Chou, D. Hogan et al., "Global, regional, and national levels and trends in maternal mortality between 1990 and 2015, with scenario-based projections to 2030: a systematic analysis by the Maternal Mortality Estimation Inter-Agency Group," *The Lancet*, vol. 387, no. 10017, pp. 462–474, 2016.
5. Abraha HE, Belachew AB, Ebrahim MM, Tequare MH, Adhana MT, Assefa NE. Magnitude, trend, and causes of under-five mortality from Kilite-Awlaelo health demographic surveillance database, northern Ethiopia, 2009–2017. BMC Public Health (2020) 20:1465, https://doi.org/10.1186/s12889-020-09554-z.
6. Y G, S B. Survival analysis of under-five mortality of children and its associated risk factors in Ethiopia. J Biosens Bioelectron. 2016; 7 (3).
7. Bryce J, Victora C, Berman P, Lawn J, Mason E, Starrs A. Fulfilling the Health Agenda for Women and Children. 2014.
8. Central Statistical Agency (CSA) [Ethiopia] and ICF. Ethiopia demographic and health survey 2016. Addis Ababa, Ethiopia, and Rockville: CSA and ICF; 2016.
9. Berhie KA, Gebresilassie HG. Logistic regression analysis on the determinants of stillbirth in Ethiopia. Maternal Health, Neonatology, and Perinatology (2016) 2:10. https://doi.org/10.1186/s40748-016-0038-5.
10. Acheampong GK, Avorgbedor YE. Determinants of under Five Mortality in Ghana; A Logistic Regression Analysis Using Evidence from the Demographic and Health Survey (1988- 2014). *American Journal of Public Health Research, 2017, Vol. 5, No. 3, 70-78.* Available online at: http://pubs.sciepub.com/ajphr/5/3/4.

11. Fenta SM, Fenta HM (2020) Risk factors of child mortality in Ethiopia: Application of multilevel two-part model. PLoS ONE 15(8):e0237640. https://doi.org/10.1371/journal.pone.0237640.
12. Farah AE, Abbas AH, Ahmed AT. Trends of admission and predictors of neonatal mortality: a hospital based retrospective cohort study in Somali region of Ethiopia. PLoS One. 2018; 13:9.
13. Desta BN, et al. Neonatal mortality and its risk factors in eastern Ethiopia: a prospective cohort study in Kersa health and demographic surveillance system (Kersa HDSS). Epidemiol Biostat Public Health. 2016; 13:4.
14. Mengesha HG, et al. Survival of neonates and predictors of their mortality in Tigray region, NorthernEthiopia. BMC Pregnancy Childbirth. 2014; 16(1):202.
15. Mekonnen Y, et al. Neonatal mortality in Ethiopia: trends and determinants. BMC Public Health. 2013; 13:483.
16. Jarso H, Workicho A, Alemseged F. Survival status and predictors of mortality in severely malnourished children admitted to Jimma University Specialized Hospital from 2010 to 2012, Jimma, Ethiopia: a retrospective longitudinal study. *BMC Pediatr*. 2015; 15:76.
17. Girum T, Kote M, Tariku B, Bekele H. Survival status and predictors of mortality among severely acute malnourished children <5 years of age admitted to stabilization centers in Gedeo Zone: a retrospective cohort study. Therapeutics and Clinical Risk Management 2017:13.
18. Desta KS. Survival status and predictors of mortality among children aged 0–59 months with severe acute malnutrition admitted to stabilization center at Sekota Hospital Waghemra Zone. *J Nutr Disord Ther*. 2015; 5:160.
19. De Maayer T, Saloojee H. Clinical outcomes of severe malnutrition in a high tuberculosis and HIV setting. *Arch Dis Child*. 2011; 96(6):560–564.
20. Lapidus N, Minetti A, Djibo A, et al. Mortality risk among children admitted in a large-scale nutritional program in Niger, 2006. *PLoS One*. 2009; 4(1):e4313.
21. Woldeamanuel BT and Gelebo KK. Statistical analysis of socioeconomic and demographic correlates of perinatal mortality in Tigray region, Ethiopia: a cross sectional study. BMC Public Health (2019) 19:1301. https://doi.org/10.1186/s12889-019-7642-z.

22. Mbiba V. Factors associated with perinatal mortality in Umguza and Bubi rural areas, 2015- the effect of maternal human immunodeficiency virus status. UNSPECIFIED: UNSPECIFIED thesis; 2015.

23. Saffron, Lale Say, João-Paulo Souza, Carol J Hogue, Dinorah L Calles, A Metin Gülmezoglu and Rosalind Raine: The relationship between maternal education and mortality among women giving birth in health care institutions: Analysis of the cross sectional WHO Global Survey on Maternal and Perinatal Health; BMC Public Health2011 11:606, https://doi.org/10.1186/1471-2458-11-60.

24. Edmond KM, Kirkwood BR, Amenga-Etego S, Owusu-Agyei S, Hurt LS. Effect of early infant feeding practices on infection-specific neonatal mortality: an investigation of the causal links with observational data from rural Ghana. Am J Clin Nutr. 2007;86(4):1126–31.

25. Bashir AO, et al. Neonatal mortality in Sudan. BMC Public Health. 2013; 13:287.

26. Zijlmans W, Hindori-Mohangoo A. Determinants of neonatal mortality in Suriname: preliminary findings from a perinatal and infant mortality survey. Ann Glob Health. 2015; 81(1):121–2.

27. Mutz-Dehbalaie, et al. Perinatal mortality and advanced maternal age. Gynecol Obstet Investig. 2014; 77(1):50–7.

28. Yaya S, Ekholuenetale M, Tudeme G, Vaibhav S, Bishwajit G, Kadio B. Prevalence and determinants of childhood mortality in Nigeria. BMC Public Health (2017) 17:485.DOI 10.1186/s12889-017-4420-7.

29. S. Yaya,G. Bishwajit, F.Okonofua, O. A.Uthman, and J. P. van Wouwe, "Under fivemortality patterns and associatedmaternal risk factors in sub-Saharan Africa: A multi-country analysis," *PLoS ONE*, vol. 13, no. 10, article no. e0205977, 2018.

30. N. S. Mugo, K. E. Agho, A. B. Zwi, E. Y. Damundu, and M. J. Dibley, "Determinants of neonatal, infant and under-five mortality in a war-affected country: analysis of the 2010 Household Health Survey in South Sudan," *BMJ Global Health*, vol. 3, no. 1, article no. e000510, 2018.

31. Berelie Y, Yismaw L, Tesfa E, Alene M.Risk factors for under-five mortality in Ethiopia: Evidence from the 2016 Ethiopian Demographic and Health Survey.*S Afr J Child Health* 2019;13(3):137-140. https://doi.org/10.7196/SAJCH.2019.v13i3.1645.

59

Additional file

Additional file 1: Table S1. Survival experience comparison

Ns	Variables	$chi^2(df)$	LRT p-value
1	Type of birth	0.07	0.798
2	Father's education level	3.1	0.213
3	Mother’s occupation	49.09	0.000*
4	Region	0.52	0.771
5	Residence	12.92	0.0003*
6	Toilet facilities	0.67	0.412
7	Mother’s education level	0.13	0.717
8	Sex of household head	22.47	0.000*
9	Sex of child	0.68	0.410
10	ANC fellow up	3.42	0.064
11	Modern contraceptive used	0.001	0.990
12	Anemic Child	25.08	0.000*
13	Exclusive breastfeeding	20.36	0.000*
14	Mother's age	22.25	0.000*
15	Child vaccinated	37.47	0.000*

LRT: Log-Rank Test.

Figures

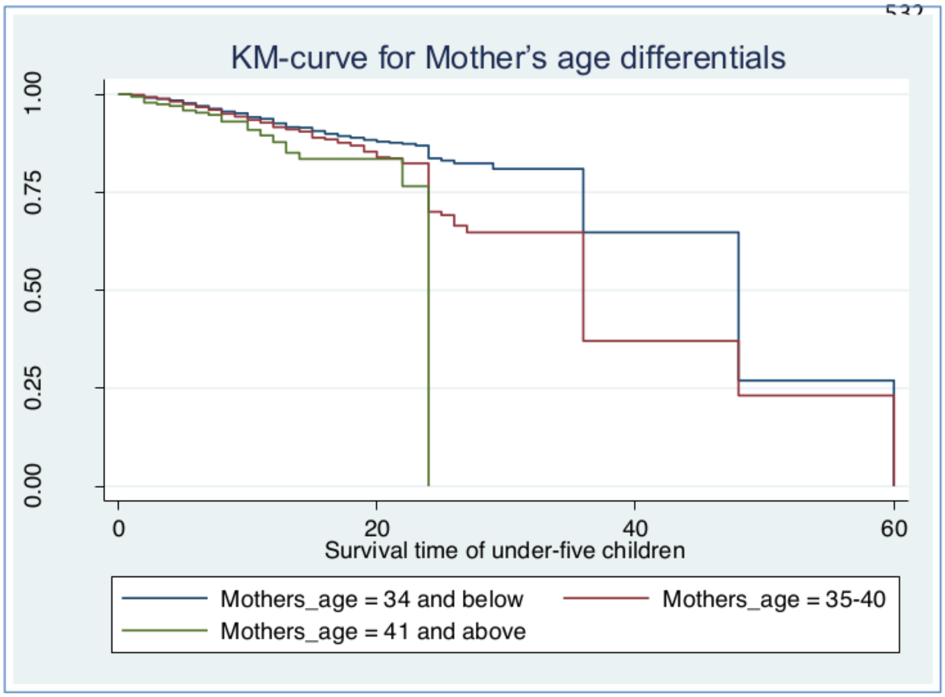

Figure. 2: Survival curve by Mothers age of children

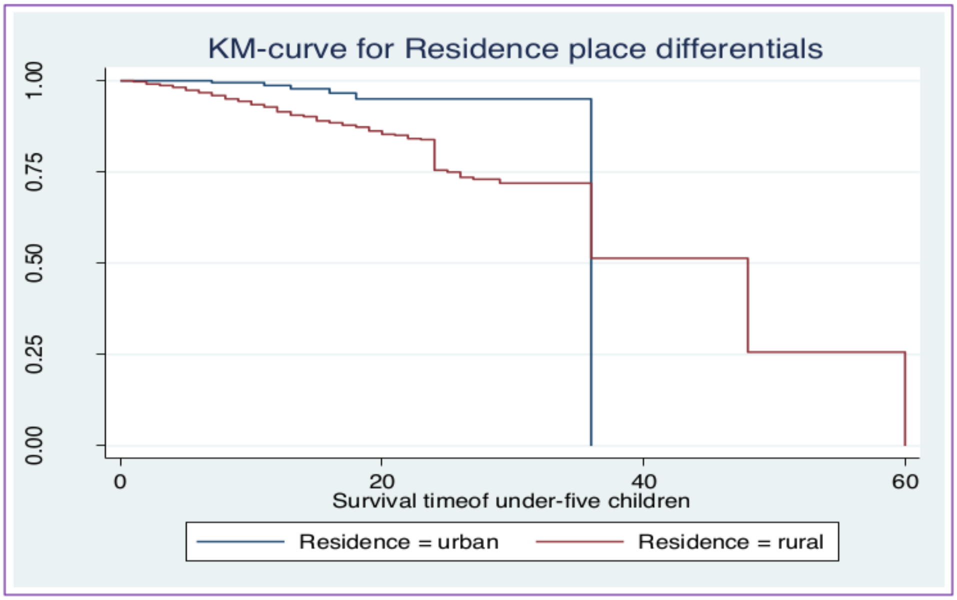

Figure. 3: Survival curve by residence place of children

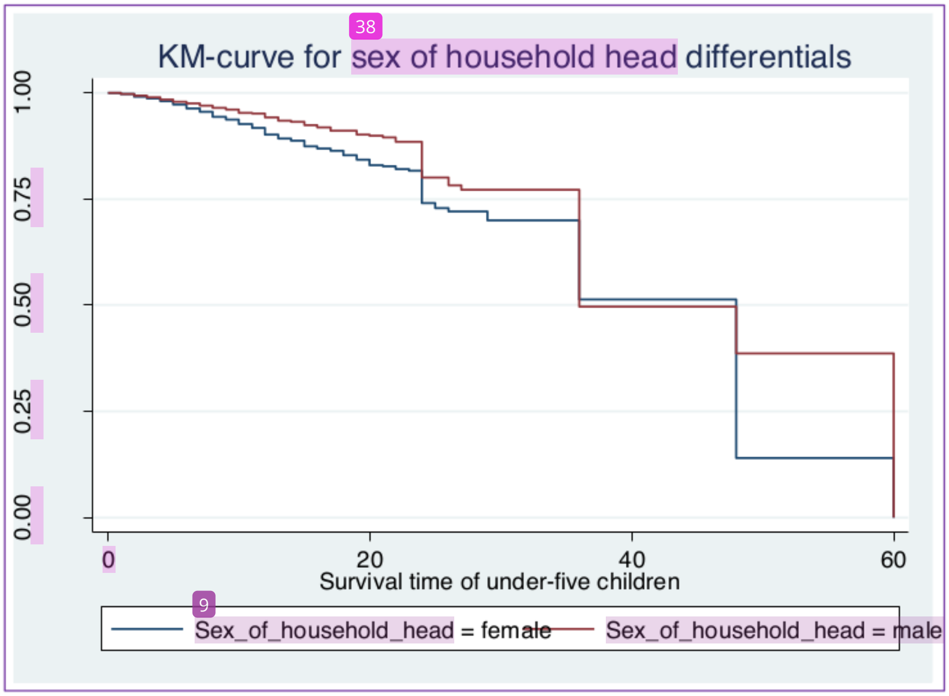

Figure. 4: Survival curve by sex of household head

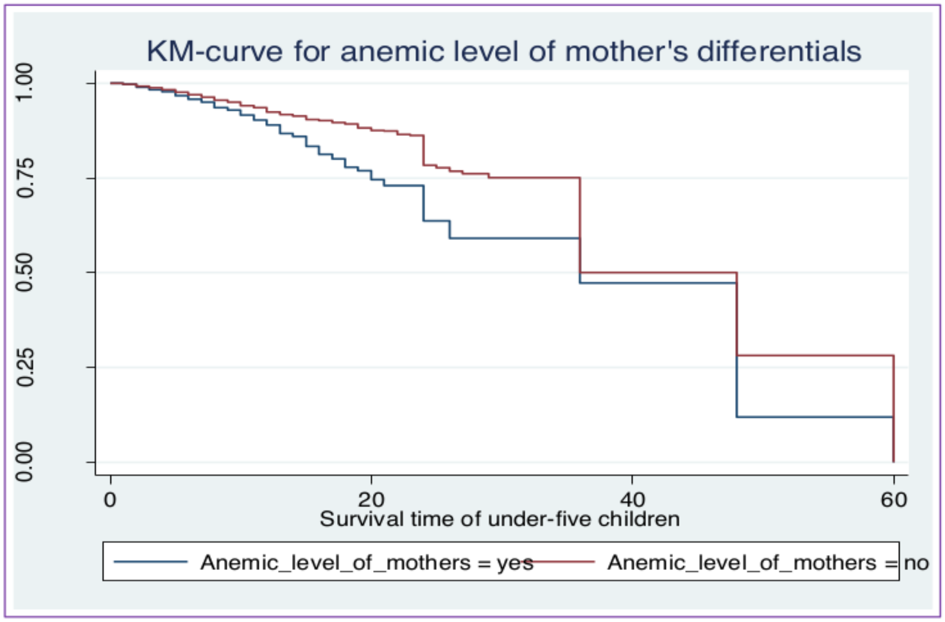

Figure. 5: Survival curve by anemic level of mother's

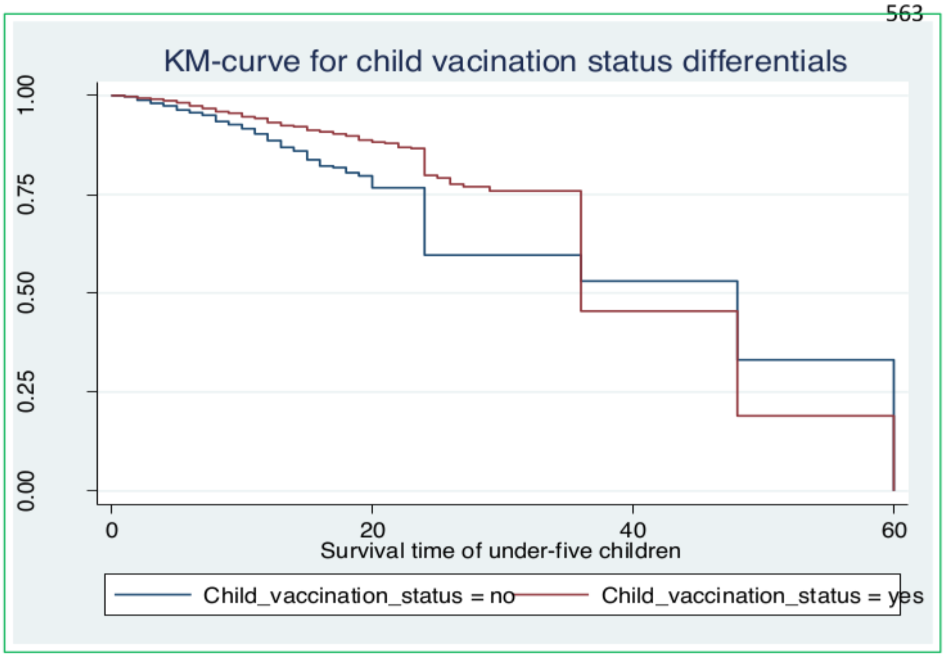

Figure. 6: Survival curve by child vaccination status

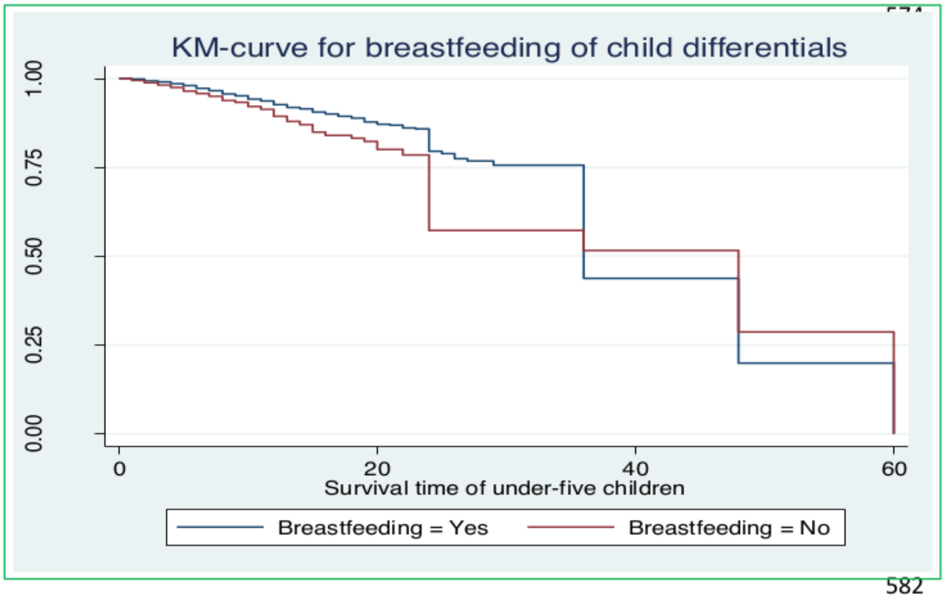

Figure. 7: Survival curve by breastfeeding of child

Permission letter and IRB approval

ICF

Mar 26, 2023

Bsrat Tesfay Hagos
Mekelle University
Ethiopia
Request Date: 03/25/2023

Dear Bsrat Tesfay Hagos:

This is to confirm that you are approved to use the following Survey Datasets for your registered research paper titled: "Time to death predictors among under-five children in pastoral regions of Ethiopia: a cross-sectional study":

Ethiopia

To access the datasets, please login at: https://www.dhsprogram.com/data/dataset_admin/login_main.cfm. The user name is the registered email address, and the password is the one selected during registration.

The IRB-approved procedures for DHS public-use datasets do not in any way allow respondents, households, or sample communities to be identified. There are no names of individuals or household addresses in the data files. The geographic identifiers only go down to the regional level (where regions are typically very large geographical areas encompassing several states/provinces). Each enumeration area (Primary Sampling Unit) has a PSU number in the data file, but the PSU numbers do not have any labels to indicate their names or locations. In surveys that collect GIS coordinates in the field, the coordinates are only for the enumeration area (EA) as a whole, and not for individual households, and the measured coordinates are randomly displaced within a large geographic area so that specific enumeration areas cannot be identified.

The DHS Data may be used only for the purpose of statistical reporting and analysis, and only for your registered research. To use the data for another purpose, a new research project must be registered. All DHS data should be treated as confidential, and no effort should be made to identify any household or individual respondent interviewed in the survey. Also, be aware that re-distribution of any DHS micro-level data, either directly or within any tool/dashboard, is not permitted. Please reference the complete terms of use at: https://dhsprogram.com/Data/terms-of-use.cfm.

The data must not be passed on to other researchers without the written consent of DHS. However, if you have coresearchers registered in your account for this research paper, you are authorized to share the data with them. All data users are required to submit an electronic copy (pdf) of any reports/publications resulting from using the DHS data files to: references@dhsprogram.com.

Sincerely,

Bridgette Wellington

Bridgette Wellington
Data Archivist
The Demographic and Health Surveys (DHS) Program

Article

ORIGINALITY REPORT

58%	56%	53%	28%
SIMILARITY INDEX	INTERNET SOURCES	PUBLICATIONS	STUDENT PAPERS

PRIMARY SOURCES

1	archpublichealth.biomedcentral.com<br>Internet Source	8%
2	Submitted to Turun yliopisto<br>Student Paper	6%
3	bmcpediatr.biomedcentral.com<br>Internet Source	4%
4	assets.researchsquare.com<br>Internet Source	4%
5	Submitted to Indiana University<br>Student Paper	4%
6	Submitted to University of Bristol<br>Student Paper	4%
7	www.researchgate.net<br>Internet Source	3%
8	www.researchsquare.com<br>Internet Source	3%
9	bmcpublichealth.biomedcentral.com<br>Internet Source	3%

10	www.hindawi.com<br>Internet Source	3%
11	link.springer.com<br>Internet Source	2%
12	journals.plos.org<br>Internet Source	2%
13	www.dovepress.com<br>Internet Source	1%
14	Submitted to Queensland University of Technology<br>Student Paper	1%
15	core.ac.uk<br>Internet Source	1%
16	bmcpregnancychildbirth.biomedcentral.com<br>Internet Source	1%
17	www.sajch.org.za<br>Internet Source	<1%
18	ouci.dntb.gov.ua<br>Internet Source	<1%
19	mhnpjournal.biomedcentral.com<br>Internet Source	<1%
20	Bsrat Tesfay Hagos, Gebru Gebremeskel Gebrerufael. "Time-to-death predictors on breast cancer patients in northern Ethiopia: a	<1%

	retrospective cross-sectional study", Research Square Platform LLC, 2023<br>Publication	
21	Submitted to University of Nigeria<br>Student Paper	<1 %
22	Sintayehu Argaw Weldemariam, Zewdu Dagnew, Yilkal Tafere, Tefera Marie Bereka, Yibelu Bazezew Bitewa. "Time todeathamong HIV-infectedunder-five children after initiation of anti-retroviral therapy and its predictors in Oromiya liyu zone, Amhara region, Ethiopia: a retrospective cohortstudy", BMC Pediatrics, 2022<br>Publication	<1 %
23	www.ncbi.nlm.nih.gov<br>Internet Source	<1 %
24	scholarworks.waldenu.edu<br>Internet Source	<1 %
25	universepg.com<br>Internet Source	<1 %
26	www.springermedizin.de<br>Internet Source	<1 %
27	Submitted to Cornell University<br>Student Paper	<1 %
28	lupinepublishers.com<br>Internet Source	<1 %

29	openpublichealthjournal.com<br>Internet Source	<1%
30	eprints.uniska-bjm.ac.id<br>Internet Source	<1%
31	jamanetwork.com<br>Internet Source	<1%
32	Lema Abate Adulo, Samuel Getachew Zewudie. "Identifying factors determining the survival time of under-five year children in rural parts of Ethiopia", Research Square Platform LLC, 2021<br>Publication	<1%
33	Walter, V., L. Jansen, M. Hoffmeister, and H. Brenner. "Smoking and survival of colorectal cancer patients: systematic review and meta-analysis", Annals of Oncology, 2014.<br>Publication	<1%
34	orcid.org<br>Internet Source	<1%
35	storage.googleapis.com<br>Internet Source	<1%
36	etd.hu.edu.et<br>Internet Source	<1%
37	Gedam Derbew Addisia, Awoke Seyoum Tegegne, Denekew Bitew Belay, Mitiku Wale Muluneh, Mahider Abere Kassaw. "Risk	<1%

Factors of White Blood Cell Progression Among Patients With Chronic Lymphocytic Leukemia at Felege Hiwot Referral Hospital, Bahir Dar, Ethiopia", Cancer Informatics, 2022
Publication

38	www.mdpi.com<br>Internet Source	<1 %
39	Submitted to Higher Education Commission Pakistan<br>Student Paper	<1 %
40	Submitted to Laureate Higher Education Group<br>Student Paper	<1 %
41	Amare Wondim, Bethelihem Tigabu, Mengistu Mekonnen Kelkay. "Time to Recovery from Severe Acute Malnutrition and Its Predictors among Admitted Children Aged 6-59 Months at the Therapeutic Feeding Center of Pawi General Hospital, Northwest Ethiopia: A Retrospective Follow-Up Study", International Journal of Pediatrics, 2020<br>Publication	<1 %
42	Getayeneh Antehunegn Tesema, Achamyeleh Birhanu Teshale, Zemenu Tadesse Tessema. "Incidence and predictors of under-five mortality in East Africa using multilevel Weibull regression modeling", Archives of Public Health, 2021	<1 %

Publication

43	Zebenay Workneh Bitew, Ermias Getaneh Ayele, Teshager Worku, Animut Alebel, Ayinalem Alemu, Frehiwot Worku, Aman Yesuf. "Determinants of mortalityamong under-five children admitted with severe acute malnutrition in Addis Ababa, Ethiopia", Nutrition Journal, 2021<br>Publication	<1 %
44	pubmed.ncbi.nlm.nih.gov<br>Internet Source	<1 %
45	Getayeneh Antehunegn Tesema, Misganaw Gebrie Worku, Achamyeleh Birhanu Teshale. "Duration of birth interval and its predictors among reproductive-age women in Ethiopia: Gompertz gamma shared frailty modeling", PLOS ONE, 2021<br>Publication	<1 %
46	Merga Dheresa, Gamachis Daraje, Gelana Fekadu, Galana Mamo Ayana et al. "Perinatal mortality and its predictors in Kersa Health and Demographic Surveillance System, Eastern Ethiopia: population-based prospective study from 2015 to 2020", BMJ Open, 2022<br>Publication	<1 %
47	www.e-epih.org<br>Internet Source	<1 %

48	2012-2017.usaid.gov<br>Internet Source	<1%
49	Getahun Dejene Yemane. "Determinant factors of under-five mortality in rural Ethiopia", Annals of Medicine and Surgery, 2022<br>Publication	<1%
50	Gizaw Sisay, Tsion Mulat. "Spatial Distribution and Determinants of Unskilled Birth Attendance in Ethiopia: Spatial and Multilevel Analysis", Research Square Platform LLC, 2023<br>Publication	<1%
51	Mark Momoh Koroma, John Alimamy Kabba, Jessicah Wanda, Jingrong Yu et al. "Under-Five Mortality in Sierra Leone and Possible Associated Factors: Evidence from the 2019 Demographic and Health Survey", Health Policy and Planning, 2022<br>Publication	<1%
52	Yonatan Eshete Ashine, Belete Achamyelew Ayele, Yared Asmare Aynalem, Getachew Yideg Yitbarek. "<br><br>Time to Death and its Predictor Among Children Under Five Years of Age with Severe Acute Malnutrition Admitted to Inpatient Stabilization Centers in North Shoa Zone, Amhara Region, Ethiopia	<1%

", Nutrition and Dietary Supplements, 2020
Publication

53 bmcresnotes.biomedcentral.com
Internet Source
<1 %

54 researchonline.lshtm.ac.uk
Internet Source
<1 %

55 Aragaw Asfaw. "SURVIVAL ANALYSIS OF UNDER-FIVE CHILDREN WITH SEVERE ACUTE MALNUTRITION IN WOLDIA HOSPITAL: RETROSPECTIVE COHORT STUDY", Indonesian Journal of Applied Research (IJAR), 2021
Publication
<1 %

56 Lema Abate Adulo, Sali Suleman Hassen, Asrat Chernet. "Timing of the First Antenatal Care Visit and Associated Risk Factors in Rural Parts of Ethiopia", International Journal of Applied Research on Public Health Management, 2022
Publication
<1 %

57 Modupe Alake Ayoade. "Spatio-temporal patterns of under 5 mortality in Nigeria", SSM - Population Health, 2018
Publication
<1 %

58 Nega Assefa, Yihune Lakew, Betelhem Belay, Haji Kedir et al. "Neonatal mortality and causes of death in Kersa Health and Demographic Surveillance System (Kersa
<1 %

HDSS), Ethiopia, 2008–2013", Maternal Health, Neonatology and Perinatology, 2016
Publication

59	bmccancer.biomedcentral.com<br>Internet Source	<1 %
60	topsecretapiaccess.dovepress.com<br>Internet Source	<1 %
61	Christian Kwaku Osei, Edward Nketiah-Amponsah. "Household Wealth Differential and Under-five Child Deaths in Ghana: Implications for Child Health Policy", Research Square Platform LLC, 2022<br>Publication	<1 %
62	Getayeneh Antehunegn Tesema, Tesfaye Hambisa Mekonnen, Achamyeleh Birhanu Teshale. "Spatial distribution and determinants of abortion among reproductive age women in Ethiopia, evidence from Ethiopian Demographic and Health Survey 2016 data: Spatial and mixed-effect analysis", PLOS ONE, 2020<br>Publication	<1 %
63	Y Berelie, L Yismaw, E Tesfa, M Alene. "Risk factors for under-five mortality in Ethiopia: Evidence from the 2016 Ethiopian Demographic and Health Survey", South African Journal of Child Health, 2019<br>Publication	<1 %

64	Animut Alebel, Fasil Wagnew, Pammla Petrucka, Cheru Tesema et al. "Neonatal mortality in the neonatal intensive care unit of Debre Markos referral hospital, Northwest Ethiopia: a prospective cohort study", BMC Pediatrics, 2020<br>Publication	<1 %
65	Berhanu Teshome Woldeamanuel, Kumachew Kusse Gelebo. "Statistical analysis of socioeconomic and demographic correlates of perinatal mortality in Tigray region, Ethiopia: a cross sectional study", BMC Public Health, 2019<br>Publication	<1 %
66	Gebru Gebremeskel Gebrerufael, Bsrat Tesfay Hagos. "Predictors of mortality among under-five children in rural Ethiopia: a cross sectional study", Research Square Platform LLC, 2023<br>Publication	<1 %
67	Mesfin Wudu Kassaw, Aele Mamo, Biruk Abate, Ayelign Kassie, Seteamlak Masresha. "High prevalence of under-five child mortality in the pastoralist communities of Ethiopia: Evidence from the 2016 Ethiopian demographic and health survey data", Research Square, 2020<br>Publication	<1 %

68	TrhasTadesse Berhe, Lebitsi Maud Modibia, Addisu Tadesse Sahile, Getachew Woldeyohanes Tedla. "Does quality of antenatal care influence antepartum stillbirth in Hossana City, South Ethiopia?", PLOS Global Public Health, 2023<br>Publication	<1%

Exclude quotes	Off	Exclude matches	Off
Exclude bibliography	Off		
