# Supplementary material for: Predictors of time to death among under-five children in pastoral regions of Ethiopia: A retrospective follow-up study
Source: PLoS One. 2024 Jul 31;19(7):e0304662. doi: 10.1371/journal.pone.0304662 (PMC11290679; doi:10.1371/journal.pone.0304662)
Supplement: Attachment — Submitted filename: Response to Reviewers.pdf [file pone.0304662.s006.pdf]

## Author’s response to reviews

**Title:** Time to death predictors among under-five children in pastoral regions of Ethiopia: a retrospective follow-up study

**Authors:**

Gebru Gebremeskel Gebrerufael (gebrugebremeskel12@gmail.com) | **ORCID**: https://orcid.org/0000-0003-2795-8529

Bsrat Tesfay Hagos (bsrattesfaygu@gmail.com) | **ORCID**: https://orcid.org/0009-0001-0487-7577

**Version:** 1 Date: 7 July 2023

**Author’s response to reviews:**

7 July 2023

**Response to editors**

Dear editors-in-chief,

The authors have enclosed a revised version of manuscript in **PLOS ONE** research article format, entitled “**Time to death predictors among under-five children in pastoral regions of Ethiopia: a retrospective follow-up study**”. In this revised version of manuscript, the authors have tried to address all of the editors’ comments, as described in detail in the Response to Editors Comments on the following pages.

As described in my cover letter accompanying this original revised version of manuscript submission, the authors believe that this revised version of manuscript fits very well within the scope of **PLOS ONE**, as it covers a number of computational approaches and issues that are dominant to the analysis of public health issues.

With warm regards,

**Bsrat Tesfay Hagos**

On the behalf of the other authors

## Response to Editor and Reviewers comments:

**Title:** Time to death predictors among under-five children in pastoral regions of Ethiopia: a retrospective follow-up study

**Authors**: Gebru Gebremeskel Gebrerufael and Bsrat Tesfay Hagos

**MS ID**: PONE-D-23-08757

**Journal**: **PLOS ONE**

**Article type**: Research article

The authors would like to thank you so much for the anonymous editors and reviewers for their precious time and understanding review of this manuscript, and for all of their supportive comments. They raise significant concerns and their inputs are very supportive for improving the main manuscript. The authors agree with almost all their comments and have revised this main manuscript accordingly. Provided underneath is a **point-by-point** response in **purple colored** describing by the authors attempts to solve all of the requested revisions in our main manuscript.

**Editor's comments:**

Journal Requirements:

When submitting your revision, we need you to address these additional requirements.

**Comment 1:** Please ensure that your manuscript meets PLOS ONE's style requirements, including those for file naming. The PLOS ONE style templates can be found at https://journals.plos.org/plosone/s/file?id=wjVg/PLOSOne_formatting_sample_main_body.pdf a nd

https://journals.plos.org/plosone/s/file?id=ba62/PLOSOne_formatting_sample_title_authors_affiliations.pdf.

**Response 1:** Thank you very much **editors**. The authors really highly appreciate for these constructive comments and the authors have followed the PLOS ONE's style requirements in order to meet its format.

**Comment 2:** PLOS requires an ORCID iD for the corresponding author in Editorial Manager on papers submitted after December 6th, 2016. Please ensure that you have an ORCID iD and that it is validated in Editorial Manager. To do this, go to 'Update my Information' (in the upper left-hand corner of the main menu), and click on the Fetch/Validate link next to the ORCID field. This will take you to the ORCID site and allow you to create a new iD or authenticate a pre-existing iD in Editorial Manager. Please see the following video for instructions on linking an ORCID iD to your Editorial Manager account: https://www.youtube.com/watch?v=_xcclfuvtxQ.

**Response 2:** Thank you so much for your insightful comments. The corresponding author creates an **ORCID**: https://orcid.org/0009-0001-0487-7577. See **on** Title page 1 and line number 11-12.

**Comment 3:** Your ethics statement should only appear in the Methods section of your manuscript. If your ethics statement is written in any section besides the Methods, please delete it from any other section.

**Response 3:** Thank you very much editor. The authors really appreciated for this valuable comment and deleted the unnecessary ethics statement which was written in the "**Declaration section**" of the main manuscript.

## **Reviewer #1**

**Comment 1:** Title is well and informative

**Response 1:** Thanks so much **Reviewer #1**. We really highly appreciate for your encouragement.

**Abstract**

**Comment 2:** Is well written, but why Italized?

**Response 2:** Thank you very much for your insightful and fruitful feedback **Reviewer #1**. The authors really appreciate for this valuable comment and have removed the unnecessary italic style according your suggestion in the main revised version of the manuscript. See Abstract section page 1 and line number 13-40.

Background

**Comment 3:** Is good, but needs some modification in logical flow and English writing improvevents

**Response 3:** Thank you so much reviewer for your perceptive and constructive comments. The authors have revised the whole part of the main revised version of the manuscript which is corrected in **purple colored**.

**Objective**

**Comment 4:** Its better if it is included in the background section as a last paragraph. In addition, it's not recommended to explain about predictors before talking about burden of the issue. Hence rearrangement is mandatory.

**Response 4:** Thank you a lot **Reviewer**, the authors moved the "**objective of the study**" in to the last paragraph of the introduction/Background section. See page 3-4 and line number 75-85.

**Method**

**Comment 5:** In lines 90, 91 "This study has used an institutionally-based retrospective study and secondary data analysis based on the 2016 Ethiopia Demographic and Health Survey (EDHS)." Indicates as the authors used two different datasets. But the analysis did not indicated it. Hence there is a methodological confussions, needs clarification for the readers

**Response 5:** Thank you so much for your insightful comments. The authors rewrite the given statement again as suggested. **See page 4 and line number 94-95.**

**Comment 6:** On line 111 & 112 "The response variable for this study was considered to be the length (survival) of time measured in months from date of birth until time-to-death (censor)", time to death is indicated as censor. But how it could be?

**Response 6:** Thank you very much reviewer. The authors revised it in the main manuscript according your suggestion. See page 6 and line number 117-120.

**Comment 7:** Table 1 (operational definitions) part is not necessary

**Response 7:** Thank you so much reviewer. The authors revised it in the main manuscript according your recommendation. See page 6 and line number 120-130.

**Results**

s**Comment 8:** Even it is good there are some points which are vegue to understand such as "Accordingly, the critical risk factors of time-to-death among children who were born to mothers with advanced age were and 2.18 times higher as compared to their reference (Adjusted Hazard Ratio (AHR) = 1.27; 95% CI: 1.06, 1.52) and (AHR = 2.18; 95% CI:1.59, 2.98), respectively."

**Response 8:** Thank you Reviewer. The authors highly appreciated your insightful comments. The authors corrected it in the main revised version of the manuscript according your constructive suggestion. See page 13 and line number 216-218.

General

**Comment 9:** What are the implications of your findings?

**Response 9:** Thank you, reviewer, for the valuable comments. The implications for these findings were already given in the "**Significance of the study**". See page 4 and line number 86-91.

**Comment 10:** What new findings you bring?

**Response 10:** **I appreciate your thoughts as well. Therefore, the new findings are given in "strength of the study".** See page 18 and line number 300-306.

**Comment 11:** Why the authors used 2016 EDHS data? Why not used the nearest dataset which is 2019?

**Response 11:** Thank you very much Reviewer. The authors highly appreciated your insightful and constructive suggestions. However, this study was conducted before the current 2019 dataset released (since 2021). At that time there was no internet connection in Tigray regional state due to the war crises between regional (Tigray) and federal government (Ethiopia). Therefore, the authors should include this in the limitation of the study. See page 18 and line number 313 - 315.

**Comment 12:** In scientific writing, its is not recommended to use pronounce such as we, our,…. So please replace with appropriate replaces.

**Response 12:** Thanks a lot reviewer. The authors highly appreciated this perceptive and fruitful suggestion. Therefore, the authors corrected the whole part on the revised version of the manuscript.

## Reviewer #2:

Dear Authors, thank you for your commitment and hard work in producing this manuscript with important research topic. I have tried to see your manuscript entitled "Time to death predictors among under-five children in pastoral regions of Ethiopia. A retrospective follow-up study "and

**Comment 1:** I found serious typo errors, significant text overlap with previously published works. Hence, it needs through edition. I will be happy if I get clarification on the following concerns:

**Response 1:** Thank you very much **Reviewer #2**. The authors highly appreciated your insightful and constructive suggestions. Therefore, the authors modified the overall parts of the revised version of the manuscript like the typo errors and overlap text of the main manuscript according your suggestions.

**Comment 2:** What is time to death? How did you determine it?

**Response 2:** Thank you very much **Reviewer #2**. The authors really appreciate for this valuable comment. Therefore, "the child's time to death, which was measured in months from birth to the end of the follow-up period, was the response variable. Events were defined as child deaths between birth and 59 months (1 = died). Children who were still alive at the end of the follow-up period and had not turned five were classified as censored (0 = censored)". See page 6 and line number 117 - 120.

**Comment 3:** Is it cross sectional or follow up study? What do you mean by institutional based retrospective cross-sectional study in abstract session? IF EDHS, so how??

**Response 3:** Thank you so much reviewer for your suggestions. Therefore, the authors corrected as your constructive suggestion. So, it is a retrospective follow-up study design which is the time from date of birth to an event, which can be measured in months and also categorized into Censored (Alive) and Death".

**Comment 4:** Was date of death recorded on the DHS data? If not how can you say time to death?

**Response 4:** Thank you very much **Reviewer**. Alright! Time from child birth to death and time from child birth to end follow-up time for alive (survival time in months) was recorded in the 2016 EDHS datasets. This, "time to death, which was measured in months from birth to the end of the follow-up period, was the response variable. Events were defined as child deaths between birth and 59 months (1 = died). Children who were still alive at the end of the follow-up period and had not turned five were classified as censored (0 = censored)".

**Comment 5:** Do you think your data is survival data? If so, why don't determine median time of death and incidence of death than prevalence? If not, how do you see using survival data analysis…cox regression, PHA…and so on?

**Response 5:** Thank you a lot **Reviewer**. Yes, it is survival data. Because it has its own retrospective follow-up time from the date of child birth until the event occurred (Dead or Censored). The mean follow-up period was 9 months. The incidence of death rate was 8.4% (95% CI 7.77%, 9.0%). See on "Abstract section" page 1 and line number 26 - 27. Moreover, this is written in the "**Result section**".

**Comment 6:** Don't you think old data to estimate the current under-five mortality? EDHS 2016???

**Response 6:** Thank you so much reviewer for your suggestions. However, this comment is already given by another reviewer and the authors have explained briefly. See on the "Limitation of the study" page 18 and line number 313 - 315.

=================================================================
