# Supplementary material for: Predictors of time to death among under-five children in pastoral regions of Ethiopia: A retrospective follow-up study
Source: PLoS One. 2024 Jul 31;19(7):e0304662. doi: 10.1371/journal.pone.0304662 (PMC11290679; doi:10.1371/journal.pone.0304662)
Supplement: Attachment — Submitted filename: Response to Reviewers_3.pdf [file pone.0304662.s008.pdf]

Author's response to reviews

Title: Time to death predictors among under-five children in pastoral regions of Ethiopia: a retrospective follow-up study

Authors:

Bsrat Tesfay Hagos (bsrattesfaygu@gmail.com) | **ORCID**: https://orcid.org/0009-0001-0487-7577

Gebru Gebremeskel Gebrerufael (gebrugebremeskel12@gmail.com) | **ORCID**: https://orcid.org/0000-0003-2795-8529

Version: 2 Date: 17-Feb-2024

Author's response to reviews:

Response to editors

Dear editors-in-chief,

The authors have been enclosed a revised version of the manuscript in **PLOS ONE** research article format, entitled "**Time to death predictors among under-five children in pastoral regions of Ethiopia: a retrospective follow-up study."** As detailed in the Response to Editors Comments on the following pages, the authors have attempted to address every one of the editors' comments in this revised version of the manuscript.

The authors feel that this revised version of the manuscript fits very well within the scope of **PLOS ONE** because it covers a number of computational approaches and issues that are important to the analysis of public health issues, as stated in my cover letter that went along with this original revised version of the manuscript submission.

With warm regards,

Bsrat Tesfay Hagos

On the behalf of the other authors

Response to Editor and Reviewers comments:

Title: Time to death predictors among under-five children in pastoral regions of Ethiopia: a retrospective follow-up study

Authors: Gebru Gebremeskel Gebrerufael and Bsrat Tesfay Hagos

MS ID: PONE-D-23-08757R1

Journal: **PLOS ONE**

Article type: Research article

The authors would like to thank you so much to the anonymous editors and reviewers for their precious time and understanding review of this manuscript and for all of their supportive and constructive comments. They raise significant concerns, and their input is very supportive of improving the main manuscript. The authors agree with almost all their comments and have revised this main manuscript accordingly. The authors provided underneath a **point-by-point** response in **purple colored,** which describes attempts to solve all of the requested revisions in our main manuscript.

Editor's comments:

Comment 1: Abstract

In the background section of the abstract, there is a controversial ideas. The statement indicated that there is a regional variation of the rate of survival among under five years children, which indicated that there are multiple studies conducted. Hence, is it beneficial to reproduce the other findings? Logical answer might be needed

Response 1: Thank you a lot. We authors have amended according your constructive comments. See on the background Abstract section.

Comment 2: Objective

According to the plos guideline, subsection of objective/s is not necessary. It is better to include in the instruction section as a last closing paragraph. Thank you authors since you considered all the comments in your revision

Response 2: Thank you very much. The authors really highly appreciate for these constructive comments and also amended according the PLOS ONE's style requirements in order to meet its format. See page 04 and line#75-79.

Comment 3: Ethical issue

I recommend attaching the consent approval letter with respect to the title as a link or other supporting information.

Response 3: Thank you so much for your insightful comments. The authors uploaded as a supplementary material about the Ethical issues.

Comment 4: Result

This is my general recommendation: please present the findings as what you found. Don't use comparative words like more, majority, …. Its better if you use them in the interpretation section(discussion).

Response 4: Thank you so much reviewer for your perceptive and constructive comments. The authors have revised the whole part of the main revised version of the manuscript which is corrected in **Page 08 and line#170-171 and 174-175**.

Comment 5: Change all P-values presented as 0.000 to 0.001

Response 5: Thank you very much. We authors have amended all over the manuscript and really highly appreciate for these constructive comments.

Comment 6: Conclusion

On line 320, the authors concluded as "*Therefore, to lower the high incidence of under-five mortality, the government should focus on the pastoral regional states of Ethiopia*", but how did you dicide that this high mortality figure is observed in only pastoral regions based on the current study?

Response 6: Thank you very much, reviewer. According to the EDHS 2016 report, the highest rate was recorded in Somalia, Benishangul-Gumuz, and Afar national regional states, where it was estimated to be about 94, 98, and 125 deaths per 1,000 live births, respectively. However, this is still higher than the average national level (Ethiopia) (i.e., reported to be 67 deaths per 1,000 live births).

General recommendation to the authors

Recommendation 1: There are grammatical errors that need dataile revision

Response 1: Thank you Reviewer. The authors highly appreciated your insightful comments. The authors have corrected it in the main revised version of the manuscript according your constructive suggestion.

Recommendation 2: Logical fallacy of some order of presentation of the data

Response 2: Thank you so much reviewer. The authors revised it in the main manuscript according your recommendation.

Recommendation 3: Better if the study was included all Ethiopian regions and use of multilevel analysis. This might have significant conclusion and greater practical implication

Response 3: Thank you very much Reviewer. The authors have highly appreciated for your impressive and constructive suggestions. See page 18 and line#316-318.

Recommendation 4: Overall the authors atached all previous recommendation, so accepted the manuscript with a minor recommendations.

Response 4: Thanks a lot reviewer. The authors have extremely respected this understanding and fruitful suggestion. Therefore, the authors corrected the whole part on the revised version of the manuscript. And also thanks so much for your encouragement.
