# Supplementary material for: Predictors of time to death among under-five children in pastoral regions of Ethiopia: A retrospective follow-up study
Source: PLoS One. 2024 Jul 31;19(7):e0304662. doi: 10.1371/journal.pone.0304662 (PMC11290679; doi:10.1371/journal.pone.0304662)
Supplement: Attachment — Submitted filename: Response to Reviewers_4.pdf [file pone.0304662.s010.pdf]

Author's response to reviews

Title: Predictors of time to death among under-five children in pastoral regions of Ethiopia: a retrospective follow-up study

Authors:

Bsrat Tesfay Hagos (bsrattesfaygu@gmail.com) | **ORCID**: https://orcid.org/0009-0001-0487-7577

Gebru Gebremeskel Gebrerufael (gebrugebremeskel12@gmail.com) | **ORCID**: https://orcid.org/0000-0003-2795-8529

Brhane Gebrehiwot Welegebrial (brhanegb008@gmail.com) | **ORCID**: https://orcid.org/0000-0003-3600-7477

Version: 3 Date: 12-April-2024

Author's response to reviews:

Response to editors

Dear editors-in-chief,

The authors have been enclosed a revised version of the manuscript in **PLOS ONE** research article format, entitled "Predictors of time to death among under-five children in pastoral regions of Ethiopia: a retrospective follow-up study" As detailed in the Response to Editors Comments on the following pages, the authors have attempted to address every one of the editors' comments in this revised version of the manuscript.

The authors feel that this revised version of the manuscript fits very well within the scope of **PLOS ONE** because it covers a number of computational approaches and issues that are important to the analysis of public health issues, as stated in my cover letter that went along with this original revised version of the manuscript submission.

With warm regards,

Bsrat Tesfay Hagos

On the behalf of the other authors

Response to Editor and Reviewers comments:

Title: Predictors of time to death among under-five children in pastoral regions of Ethiopia: a retrospective follow-up study

Authors: Gebru Gebremeskel Gebrerufael and Bsrat Tesfay Hagos

MS ID: PONE-D-23-08757R1

Journal: **PLOS ONE**

Article type: Research article

The authors would like to thank you so much to the anonymous editors and reviewers for their precious time and understanding review of this manuscript and for all of their supportive and constructive comments. They raise significant concerns, and their input is very supportive of improving the main manuscript. The authors agree with almost all their comments and have revised this main manuscript accordingly. The authors provided underneath a **point-by-point** response in **purple colored,** which describes attempts to solve all of the requested revisions in our main manuscript.

<u>Editor's comments:</u>

Comment 1: Please modify Title of the manuscript **"Time to death predictors among under-five children in pastoral regions of Ethiopia: a retrospective follow-up study" in to the form of "**Predictors of time to death among under-five children in pastoral regions of Ethiopia: a retrospective follow-up study**"**

Response 1: Thank you very much Reviewer. The authors have really highly appreciated for these constructive comments and also amended according your best suggestion. See page #01 and line #01-02.

Comment 2:

Email addresses:

GGG: gebrugebremeskel12@gmail.com | **ORCID**: https://orcid.org/0000-0003-2795-8529

ORCID: https://orcid.org/0000-0003-2795-8529

BTH: bsrattesfaygu@gmail.com(*corresponding author) | **ORCID**: https://orcid.org/0009-0001-0487-7577

Please change into the form of this "**Correspondence: BTH:** bsrattesfaygu@gmail.com"

Response 2: Thank you Reviewer. The authors have really highly appreciated these suggestion and constructive comments. We authors modified according your constructive comments. **See page #01 and line #10.**

Comment 3: Abstract section

Methods: Between January 18 and June 27, 2016, a retrospective follow-up study design was done on under-five children in Ethiopia's pastoral areas. **Please change this in to the form of the "Methods:** Between January 18 and June 27, 2016, a retrospective follow-up study design was done among under-five children in pastoral areas of Ethiopia".

Response 3: Thank you a lot Reviewer. We authors modified according your constructive comments. **See page #01 and line 19-20.**

Comment 4: Abstract section of the "**Result"**

Results: A total sample size of 7,677 was selected.

Response 4: Thank you a lot Reviewer. We authors modified according your constructive comments. **See page #02 and line #25.**

Comment 5: on the "**Result"** section, what does this mean? Advanced mothers age?

Response 5: Thank you a lot Reviewer. We authors modified according your constructive comments. **See page #2 and line #27.**

Comment 6: on the "**Result"** section, you mean not initiating EBF early or no EBF at all

Author Response 6: Yes. Thank you very much **Reviewer** you right. We authors highly appreciate for your encouragement.

Comment 7: on the "**Result"** section, is that being farmer or being agricultural sector professional? Much vague

Author Response 7: Thank you a lot! We authors mean that those persons whose economic activities have done using agricultural activities.

Comment 8: On the "**Result"** section, were "discovered to be highly reliable indicators of under-five children's time to death".

Author Response 8: Thank you so much reviewer for your constructive and impressive suggestions. Therefore, the authors corrected according your suggestions. See on the **page #02 and line #32 - 33.**

Comment 9: on the "**Whole manuscript"** section**:** … very poor language write up, and it is typical to the overall manuscript content. Please revise your manuscript thoroughly for language.

Author Response 9: Thank you so much reviewer for your perceptive and constructive comments. The authors have revised the whole part of the main revised version of the manuscript which is corrected it.

Comment 10: on the "**Introduction"** section**:** Please revise the introduction section with more relevant and upto-date information. Focus on your main objective which is the predictors of malnutrition and summarize the implication of available.

Author Response 10: Thank you very much. The authors really highly appreciate for these constructive comments and also amended according to your comment. See on the **page #02 and line #43 - 44.**

Comment 11: The majority of low- and middle-income nations had not yet achieved the fourth Millennium Development Goal (MDG4), which called for a 67% reduction in under-five mortality [7]. Please remove this and better to use the SDG as reference indicator not the outdated MDG.

Author Response 11: Thank you a lot. We authors have amended according your constructive comments. See on Page #03 and line#59-62.

Comment 12: "antenatal care (ANC) fellow"

Author Response 12: Thanks for your constructive comment. We authors mean that the "ANC fellow-up" is visit of mothers to be healthy persons.

Comment 13: "**Objectives of the study**"

General objective

This study's overarching goal was to identify the potential risk factors that led to a shorter survival time-to-death for children under the age of five in pastoral regions of Ethiopia.

Specific objectives

➢To determine the incidence of mortality among under-five children in the pastoral regions of Ethiopia

➢To identify predictors associated with time-to-death among under-five children in the pastoral regions of Ethiopia

➢To estimate the survival time and compare survival curves between the different categories of predictors associated with time-to-death among under-five children…………………

"This should be summarized in one sentence as the last sentence in the introduction section. Also the contents of your manuscript is different from the one on the system with the corrections (track change)"

Author Response 13: Thank you a lot! We authors corrected according your suggestions.

Comment 14: "**Significance of the study**"

The findings of this study will help develop initiatives to increase the survival time of children under the age of five in the study area and other Ethiopian regions, and the data from this study will be used as a starting point for future research. Additionally, this study may shed light on the most effective measures to lower under-five mortality for healthcare professionals, decision-makers, and society at large.

Author Response 14: Thanks for your constructive comment. We authors amended according your comments. See on the **page #3 and line #80 - 84.**

Comment 15: Please change the word "**Operational definitions**" **in to "Measurements"**

Author Response 15: Thank you so much reviewer for your constructive suggestions. Therefore, the authors corrected according your suggestion. See on the **page #7 and line #150.**

Comment 16: Your **Table 1** formats are also not to the standard. Correct as it was done on the first variable. Too long table also

Author Response 16: Thank you so much reviewer for your constructive suggestions. Consequently, the authors have corrected and make it short according your suggestion. See on **Table 1** on the **page #8 and line #176 - 178.**
