# Supplementary material for: Predictors of time to death among under-five children in pastoral regions of Ethiopia: A retrospective follow-up study
Source: PLoS One. 2024 Jul 31;19(7):e0304662. doi: 10.1371/journal.pone.0304662 (PMC11290679; doi:10.1371/journal.pone.0304662)
Supplement: Attachment — Submitted filename: comments.docx [file pone.0304662.s004.docx]

Time to death predictors among under-five children in pastoral regions of Ethiopia: a cross-sectional study

Title is well and informative

Abstract

Is well written, but why Italized?

Background

Is good, but needs some modification in logical flow and English writing improvevents

Objective

Its better if it is included in the background section as a last paragraph

In addition, its not recommended to explain about predictors before talking about burden of the issue. Hence rearrangement is mandatory

Method

In lines 90,91 “This study has used an institutionally-based retrospective study and secondary data analysis based on the 2016 Ethiopia Demographic and Health Survey (EDHS).” Indicates as the authors used two different datasets. But the analysis did not indicated it

Hence there is a methodological confussions, needs clarification for the readers

On line 111 & 112 “The response variable for this study was considered to be the length (survival) of time measured in months from date of birth until time-to-death (censor)”, time to death is indicated as censor. But how it could be?

Table 1 (operational definitions) part is not necessary

Results

Even it is good there are some points which are vegue to understand such as “Accordingly, the critical risk factors of time-to-death among children who were born to mothers with advanced age were and 2.18 times higher as compared to their reference (Adjusted Hazard Ratio (AHR) = 1.27; 95% CI: 1.06, 1.52) and (AHR = 2.18; 95% CI:1.59, 2.98), respectively.”

General

What is the implications of your findings?

What new findings you bring?

Why the authors used 2016 EDHS data? Why not used the nearest dataset which is 2019?

In scientific writing, its is not recommended to use pronounce such as we, our,…. So please replace with appropriate replaces.
