# Supplementary material for: Predictors of time to death among under-five children in pastoral regions of Ethiopia: A retrospective follow-up study
Source: PLoS One. 2024 Jul 31;19(7):e0304662. doi: 10.1371/journal.pone.0304662 (PMC11290679; doi:10.1371/journal.pone.0304662)
Supplement: Attachment — Submitted filename: Comment on Time to death predictors among under.docx [file pone.0304662.s007.docx]

Comment on **Time to death predictors among under-five children in pastoral regions of Ethiopia: a retrospective follow-up study**

**Abstract**

**In the background section of the abstract, there is a controversial ideas. The statement indicated that there is a regional variation of the rate of survival among under five years children, which indicated that there are multiple studies conducted. Hence, is it beneficial to reproduce the other findings? Logical answer might be nneded**

**Objective**

**According to the plos guideline, subsection of objective/s is not necessary. It is better to include in the instruction section as a last closing paragraph. Thank you authors since you considered all the comments in your revision**

**Ethical issue**

**I recommend to attach the consent approval letter with respect to the title as a link or other supporting information.**

**Result**

**This is my general recommendation: please present the findings as what you found. Don’t use comparative words like more, majority, …. Its better if you use them in the interpretation section(discussion).**

**Change all P-values presented as 0.000 to 0.001**

**Conclusion**

**On line 320, the authors concluded as “*Therefore, to lower the high incidence of under-five mortality, the government should focus on the pastoral regional states of Ethiopia*” , but how did you dicide that this high mortality figure is observed in only pastoral regions based on the current study?**

**General recommendation to the aouthros**

1. **There are grammatical errors that need dataile revision**
2. **Logical fallacy of some order of presentation of the data**
3. **Better if the study was included all Ethiopian regions and use of multilevel analysis. This might have significan conclusion and greater practical implication**
4. **Overall the authors tached all previous recommendation, so accepted the manuscript with a minor recommendations.**
