# Supplementary material for: Predictors of time to death among under-five children in pastoral regions of Ethiopia: A retrospective follow-up study
Source: PLoS One. 2024 Jul 31;19(7):e0304662. doi: 10.1371/journal.pone.0304662 (PMC11290679; doi:10.1371/journal.pone.0304662)
Supplement: Attachment — Submitted filename: Manuscript_3 Editor comments.docx [file pone.0304662.s009.docx]

**Predictors of time to death among under-five children in pastoral regions of Ethiopia: a retrospective follow-up** **study**

Gebru Gebremeskel Gebrerufael^1¶^ and Bsrat Tesfay Hagos^2*¶^

^1^Department of Statistics, College of Natural and Computational Science, Adigrat University, P.O. Box 50, Adigrat, Ethiopia

^2^Department of Statistics, College of Natural and Computational Science, Mekelle University, P.O. Box 231, Mekelle, Ethiopia

Correspondence: **BH:** [bsrattesfaygu@gmail.com](mailto:bsrattesfaygu@gmail.com) (BTH)

**Abstract**

**Background:** Although the mortality rate for children under five has significantly declined globally over time, Sub-Saharan African nations, particularly Ethiopia, continue to bear a disproportionately large amount of this burden. Under-five child mortality in Ethiopia showed a significant decline over time, but the degree of decline differed across regional states. Therefore, the goal of this study was to identify the key factors that contributed to a shorter survival time to death for children under the age of five in Ethiopia's pastoral regions.

**Methods:** Between January 18 and June 27, 2016, a retrospective follow-up study design was done among under-five children in pastoral areas of Ethiopia.

The log-rank test was performed to demonstrate the statistically significant difference between categorical predictors, and the Kaplan-Meier survival curve was utilized to estimate survival time. The Cox proportional hazards (PH) model analyses of bivariable and multivariable variables were fitted to find time-to-death predictor factors in under-five children.

**Results:** A total 7,677 children were included in the study. The overall incidence rate of under-five mortality was 8.4% (95% CI 7.77%, 9.0%). In the multivariable Cox PH model analysis, children vaccinated (AHR: 0.72, 95% CI: 0.59, 0.88), advanced mothers aged 35–40 (AHR: 1.27; 95% CI: 1.06, 1.52), and above 41 (AHR: 2.18, 95% CI: 1.59, 2.98), not initiating exclusively breastfeeding (AHR: 1.26, 95% CI: 1.02, 1.55), the agriculture sector of the mother’s occupation (AHR: 2.57, 95% CI: 1.74, 3.31), the male sex of the household head (AHR: 0.67, 95% CI: 0.56, 0.81), non-anemic Child (AHR: 0.67, 95% CI: 0.55, 0.83), and rural residence (AHR: 3.27, 95% CI: 1.45, 7.38) were identified as main predictors of time to death among under-five children.

**Conclusions:** In this study, the authors found a higher rate of under-five deaths than the national figure. A child vaccinated, exclusively breastfeeding, mother’s occupation, sex of household head, anemic Child, mother's age, and residence were found to be the most influential predictors for time-to-death. Therefore, to lower the high incidence of under-five mortality, the government should focus on the pastoral regional states of Ethiopia.

**Keywords:** Time to death, Under-five children mortality, Cox PH model, Pastoral regions

**Introduction**

The likelihood that a child born in a given year would pass away before turning five is known as the under-5 child mortality rate (U5CMR). Children's intellectual and physical development is crucial throughout the first five years of life [1].

Although the U5CMR has decreased globally, from 5.9 million child deaths in 2015 to 5.3 million in 2018, there is still a high mortality rate in African nations, with Ethiopia having the highest rate at nearly one-twelfth (81 deaths per 1,000 live births), which is roughly seven times higher than in European nations [1, 2].

More than 25% of all under-five-year-old child deaths worldwide take place in Africa [3]. Somalia (133/1000 live births), Chad (127/1000 live births), the Central African Republic (124/1000 live births), Sierra Leone (114/1000 live births), Mali (111/1000 live births), and Nigeria (104/1000 live births) were the top six African nations with the highest U5CMR in 2016, accounting for more than 50% of under-five mortality in Sub-Saharan African (SSA) countries and roughly 1/5 of under-five mortality globally [3].

With one child out of every eleven live births dying before the age of five, the SSA continues to have the highest U5CMR in the world [4, 5]. So, the death of children under the age of five continues to be a major public health issue in the SSA [5]. Similar to this, U5CMR in Ethiopia is one of the most significant and challenging public health issues that needs to be highlighted [6].

The majority of low- and middle-income nations had not yet achieved the fourth Millennium Development Goal (MDG4), which called for a 67% reduction in under-five mortality [7].

With Ethiopia's current child mortality rates, it will be difficult to achieve the 2030 goal of reducing the U5CMR to 55 deaths per 1,000 live births, and research has shown that there is a significant regional difference in under-five mortality [6]. The highest rate was recorded in Somalia, Benishangul-Gumuz, and Afar national regional states, where it was estimated to be about 94, 98, and 125 deaths per 1,000 live births, respectively [8]. This means that the U5CMR in Ethiopia is still higher (i.e., reported to be 67 deaths per 1,000 live births). Additionally, research from many sources showed that among the predictor factors linked to U5CMR were anemic children, poor mother education level, failure to initiate breastfeeding, antenatal care (ANC) fellows, advanced mother age, and unvaccinated children [1, 3, 9–11]. To alleviate these problems, Ethiopia is one of the SSA nations working to reduce U5CMR. The government has implemented various interventions to improve coverage, quality, and use of skilled care, as well as important newborn care and management of preterm and low birth weight. However, it continued to be quite high in Ethiopia's pastoral regions, including Somalia, Benishangul-Gumuz, and Afar [7, 8].

**Objectives of the study**

**General objective**

This study's overarching goal was to identify the potential risk factors that led to a shorter survival time-to-death for children under the age of five in pastoral regions of Ethiopia.

**Specific objectives**

➢To determine the incidence of mortality among under-five children in the pastoral regions of Ethiopia

➢To identify predictors associated with time-to-death among under-five children in the pastoral regions of Ethiopia

➢To estimate the survival time and compare survival curves between the different categories of predictors associated with time-to-death among under-five children.

**Significance of the study**

The findings of this study will help develop initiatives to increase the survival time of children under the age of five in the study area and other Ethiopian regions, and the data from this study will be used as a starting point for future research. Additionally, this study may shed light on the most effective measures to lower under-five mortality for healthcare professionals, decision-makers, and society at large.

**Materials and methods**

**Study design, source of data, and period**

This retrospective follow-up study design was conducted using secondary data analysis based on the 2016 Ethiopia Demographic and Health Survey (EDHS) data. A nationally representative sample of roughly 16,650 homes was chosen for the 2016 EDHS, which was carried out with the cooperation of the Ministry of Health (MOH) and carried out by the Central Statistical Agency (CSA) and partner organizations between January 18, 2016, and June 27, 2016. Individual interviews were available with all of the men and women in these families who were of reproductive age (15–49 and 15–59, respectively).

**Sampling technique, inclusion, and exclusion criteria**

A stratified sample design with two stages was used by the 2016 EDHS. Nine regional states and two administrative cities make up the nation. 443 rural and 202 urban enumeration areas were chosen at random in the first round, with the probability proportionate to size. In the second stage, a cluster of equal probability and systematic selection from the freshly compiled household list involved 28 homes. The regional states of Afar, Benishangul-Gumuz, and Somalia were chosen because, according to the 2016 EDHS data, they have the highest U5CMR. In these areas, a total sample of 7,677 women with live births in the five years before the survey (642 occurrences and 7,035 censored children) was included for this study's analysis. The detailed diagrammatic sample selection procedure was presented (see **Figure 1**).

Figure 1: Diagrammatic presentation of sample selection among under-five children included in this study, 2016

**Study variables**

**Response variable**

The child's time to death, which was measured in months from birth to the end of the follow-up period, was the response variable. Events were defined as child deaths between birth and 59 months (1 = died). Children who were still alive at the end of the follow-up period and had not turned five were classified as censored (0 = censored).

**Predictor variables**

The primary factors connected with time-to-death among children under the age of five are type of birth child’s (single, multiple), father's education level (no education, primary, secondary and above), mother’s occupational (not working, non-agriculture sector, agriculture sector), region (Affar, Somalia, Benshangul-gumuaz), residence (urban, rural), toilet facilities (no facility, with facility), mother’s education level (no education, primary and above), sex of household head (female, male), sex of child (male, female), ante-natal care (ANC) fellow-up (no, yes ), modern contraceptive method used (no, yes), anemic child (yes, no), exclusively breastfeeding (yes, no), mother's age (in year) (34 and below, 35–40, 41 and above), and child vaccinated (no, yes); they were chosen for this investigation from the available similar studies on the topic.

**Statistical methods of data analysis**

In the bivariable STATA software version 14 package (i.e., the data was extracted, recorded, and analyzed). To explore all of the study's variables, descriptive statistics, including frequency distributions and percentages, were used. For categorical predictor variables, the Kaplan-Meier (K-M) estimators were used to display the survival experience of children through time. In order to determine whether there is a statistically significant difference between the predictor variables contributing to the ability of under-five children to survive, the survival curves were compared and tested using the log-rank test (see **Additional file 1: Table S1**). The Cox proportional hazards (PH) model is one of the regression models used in survival analysis most frequently. Modeling Cox proportional hazard (PH),

${\begin{aligned} . \\ h\left( t \right)=h \end{aligned}}_{0}\left( t \right)*{exp}^{(b_{1}x_{1}{+ b}_{2}x_{2} + b_{3}x_{3} + \ldots\ldots\ldots\ldots.. b_{p}x_{p})}$ were used to identify and to check the impact of each independent variables on the time-to-death. The hazard function $h\left( t \right)$ is determined by a set of p covariates ($x_{1}$,$x_{2}, x_{3}$, ……,$x_{p}$), whose impact is measured by the size of the respective coefficients ($b_{1}$,$b_{2}, b_{3}$,……,$b_{p}$). The term $h_{0}$(t) is called the baseline hazard for a time-to-death. All predictor variables and the overall proportionality test (Shoenfield residual test) provided evidence in support of the proportionality assumptions of the Cox PH regression model analysis. Each predictor variable's bivariable Cox PH regression model was fitted once the proportional hazard assumption had been verified.

Furthermore, those variables with a p-value ≤ 0.25 in the bivariable model analysis were included in the multivariable Cox PH regression model. Crude and adjusted hazard ratios with a 95% Confidence Interval (CI) were utilized to examine statistically significant factors and gauge the strength of associations. In the multivariable Cox PH regression model analysis, variables with a P-value < 0.05 were discovered to be significant indicators of time to death in children under the age of five. The Likelihood Ratio Test (LRT) was used to evaluate the final model's goodness of fit.

**Measurements**

In this study, "**survival time**" was defined as the period from the commencement of observation to the occurrence of the observation's result (event or suppression).

**Event:** In this study, the event referred to the study subject (1) who had died during the observation period and had experienced the event outcome.

**Censored:** The study of children under the age of five who were still alive at the end of the follow-up is referred to as censored.

**Ethical consideration**

In order to use the 2016 EDHS dataset from the DHS program for the current study, a formal consent letter was discovered. The dataset was also given IRB approval for public use without individual or household identity. As a result, the participants' identities were kept secret. Furthermore, this dataset was exclusively used for the current study in accordance with the DHS program discipline, rules, and regulations. However, access to the study's data set requires legal registration at <https://dhsprogram.com/data/available-datasets.cfm> and the creation of a persuasive letter outlining the project's descriptive goal for the DHS program.

**Results**

**Descriptive statistics**

In this study, a total sample of 7,677 was selected for analysis, with 642 under-five deaths. The majority (96.6%) and (84.7%) of the study participants lived in rural areas and had no education level, respectively. Approximately 98.6% of study participants were single-birth types, and 49.2% were from the Somali regional state. About 79.1% of respondents had toilet facilities, and the majorities (64.2%) of participants were female household heads. Concerning the use of the modern contraceptive method, 94.1% of respondents did not use the modern contraceptive method.

Most (75.6%) fathers have no education level, and 64.3% of mothers did not have ANC follow-up during their last pregnancy. Moreover, 51.9% of children were male, and the majorities (79.1%) of children were not anemic (**Table 1**).

**Table 1:** Mother’s and child’s socio-demographic characteristics, health, and environmental predictors in the pastoral regions of Ethiopia, 2016 (N = 7,677)

| Variables |  | Frequency (N) | Percentage (%) |
| --- | --- | --- | --- |
| Type of birth | Single | 7572 | 98.6 |
|  | Multiple | 105 | 1.4 |
| Father's education level |  |  |  |
| No education |  | 5803 | 75.6 |
| Primary | | 1086 | 14.1 |
| Secondary and above | | 788 | 10.3 |
| Mother’s occupation | |  |  |
| Not working | | 5722 | 74.5 |
| Non-agriculture sector | | 1680 | 21.9 |
| Agriculture sector | | 275 | 3.6 |
| Region | |  |  |
| Affar | | 2287 | 29.8 |
| Somalia | | 3775 | 49.2 |
| Benshangul-gumuaz | | 1615 | 21 |
| Residence | |  |  |
| Urban | | 261 | 3.4 |
| Rural | | 7416 | 96.6 |
| Toilet facilities | |  |  |
| No facility | | 1606 | 20.9 |
| With facility | | 6071 | 79.1 |
| Mother’s education level | |  |  |
| No education | | 6502 | 84.7 |
| Primary and above | | 1175 | 15.3 |
| Sex of household head | |  |  |
| Female | | 4931 | 64.2 |
| Male | | 2746 | 35.8 |
| Sex of child | |  |  |
| Male | | 3986 | 51.9 |
| Female | | 3691 | 48.1 |
| ANC fellow-up | |  |  |
| No | | 4937 | 64.3 |
| Yes | | 2740 | 35.7 |
| Modern contraceptive used | |  |  |
| No | | 7224 | 94.1 |
| Yes | | 453 | 5.9 |
| Anemic Child | |  |  |
| Yes | | 1605 | 20.9 |
| No | | 6072 | 79.1 |
| Exclusively breastfeeding | |  |  |
| Yes | | 4738 | 61.7 |
| No | | 2939 | 38.3 |
| Mother's age | |  |  |
| 34 and below | | 3363 | 43.8 |
| 35–40 | | 3780 | 49.2 |
| 41 and above | | 534 | 7 |
| Child vaccinated | |  |  |
| No | | 3058 | 39.8 |
| Yes | | 4619 | 60.2 |

**Comparison of the survival ability of under-five children**

Along with the descriptive data shown in **Table 2** above, Kaplan-Meier (K-M) survival curve estimators are shown in **Figures 2–7** for the most important predictor variables. These samples of K-M curves show that under-five-years-old children whose advanced mother’s age, rural residence, being male sex household head, being anemic, not vaccinated, and not initiating exclusive breastfeeding had shorter survival times compared to those reference categories. Additionally, from the log-rank test, there was a significant difference between the mother’s age (P-value = 0.000), residence groups (P-value = 0.000), sex of household head (P-value = 0.000), anemic Child (P-value = 0.000), vaccinated Child (P-value = 0.000), and exclusive breastfeeding (P-value = 0.000) on the time-to-death among under-five children (see **Additional file 1: Table S1**).

**Time-to-death predictors among under-five children**

According to this study, there were 8.4 deaths per 100 live births over the study period as a result of U5CMR generally. The mean follow-up period was 9 months. None of the predictors violated the Cox PH regression model's essential assumption (see **Table 2**).

**Table 2:** Test of proportional-hazards assumption (STATA software version 14)

| Variables | Rho | Chi^2^ | P-value |
| --- | --- | --- | --- |
| Type of birth (reff.= single) |  |  |  |
| Multiple | -0.024 | 0.3 | 0.5808 |
| Father's education level (reff.= no education) |  |  |  |
| Primary | 0.0537 | 1.46 | 0.2275 |
| Secondary and above | -0.024 | 0.29 | 0.5923 |
| Mother’s occupation (reff.= not working) |  |  |  |
| Non-agriculture sector | 0.07202 | 2.76 | 0.0964 |
| Agriculture sector | -0.0201 | 0.23 | 0.6347 |
| Region (reff.= Affar) |  |  |  |
| Somalia | -0.01 | 0.06 | 0.8087 |
| Benshangul-gumuaz | -0.028 | 0.48 | 0.4884 |
| Residence (reff.= urban) |  |  |  |
| Rural | -0.068 | 2.68 | 0.1018 |
| Toilet facilities (reff.= no facility) |  |  |  |
| With facility | 0.029 | 0.55 | 0.4597 |
| Mother’s education level (reff.= no education) |  |  |  |
| Primary and above | -0.047 | 1.23 | 0.2675 |
| Sex of household head (reff.= female) |  |  |  |
| Male | -0.006 | 0.02 | 0.8878 |
| Sex of child (reff.= male) |  |  |  |
| Female | -0.04 | 0.86 | 0.3527 |
| ANC fellow up (reff.= no ) |  |  |  |
| Yes | 0.0291 | 0.48 | 0.4876 |
| Modern contraceptive used (reff.= no) |  |  |  |
| Yes | -0.036 | 0.75 | 0.3874 |
| Anemic Child (reff.= yes) |  |  |  |
| No | -0.023 | 0.28 | 0.5944 |
| Exclusively breastfeeding (reff.= yes) |  |  |  |
| No | -0.033 | 0.62 | 0.4321 |
| Mother's age (reff.= 34 and below) |  |  |  |
| 35–40 | 0.0744 | 3.02 | 0.0822 |
| 41 and above | 0.04422 | 1.07 | 0.3019 |
| Child vaccinated (reff.= no) |  |  |  |
| Yes | 0.03674 | 0.76 | 0.3832 |
| Global test | ------- | 18.56 | 0.4854 |
| **N.B:** Chisq refers to chi-square statistic value; Rho refers to the correlation among covariates and survival times. | | | |

Significant predictors of U5CMR in the bivariable Cox PH regression model study (p-value ≤ 0.25): type of birth, father's education level, region, mother's occupation, residence, toilet facilities, mother’s education level, sex of household head, sex of the child, ANC fellow-up, modern contraceptives used, anemic Child, exclusively breastfeeding, mother's age, and Child vaccinated. Only eight variables were shown to be highly significant predictors of time to death in under-five-year-old children in the multivariable Cox PH regression model study. As a result, the adjusted hazard ratios (AHR) for children born to mothers who were older than average were 1.27 and 2.18 times higher than those for children born to younger mothers (AHR = 1.27; 95% CI: 1.06, 1.52) and (AHR = 2.18; 95% CI: 1.59, 2.98), respectively. The risk of death for children born to mothers who worked in the agricultural sector was 2.57 times higher than the risk for children born to mothers who did not work (AHR: 2.57; 95% CI: 1.74, 3.31). Children from a male household head had under-five mortality rates that were nearly 33% lower than those from a female household head (AHR: 0.67; 95% CI: 0.56, 0.81) for the variable sex of household head. Children who had not been anemic were 33% less likely to die (AHR: 0.67; 95% CI: 0.55, 0.83) than children who had been anemic. Additionally, compared to children who exclusively breastfed from the beginning, children who did not were 1.26 times (AHR: 1.26; 95% CI: 1.02, 1.55) more likely to pass away. Furthermore, a strong correlation between child immunization and time to death in children under the age of five was discovered. Children who received vaccinations had under-five time-to-death risks that were 38% (AHR: 0.72; 95% CI: 0.59, 0.88) lower than children who did not receive vaccinations. Finally, the residency had a negative effect on the time to death for children under the age of five. Children whose mothers resided in rural areas had 3.27 times (AHR: 3.27; 95% CI: 1.45, 7.38) greater mortality rates than children whose mothers resided in urban areas (**Table 3**).

**Cox PH regression model and model adequacy checking results**

A study of the Cox PH regression model was used to determine the impact of each covariate on the time until a child under the age of five died. The Likelihood Ratio Test (LRT) of the Cox PH regression model analysis with the Breslow method was used to test model adequacy. The test statistic was 142.32 with a *p*-value of 0.000, which showed that the model is a good fit for the data set at a 5% level of significance (**Table 3**).

**Table 3:** Bivariable and multivariable Cox PH regression model analysis of time-to-death predictors among under-five children in the pastoral regions of Ethiopia, 2016 (N = 7,677)

| Variables | Survival status | | CHR (95% CI) | AHR (95% CI) | P-value |
| --- | --- | --- | --- | --- | --- |
|  | Censored | Event |  |  |  |
| Mother’s occupation |  |  |  |  |  |
| Not working (reff.) | 5263 | 459 | 1 | 1 |  |
| Non-agriculture sector | 1547 | 133 | 0.64 (0.52, 0.8)* | 0.78 (0.63, 0.98)* | 0.036* |
| Agriculture sector | 225 | 50 | 2.1 (1.54, 2.86)* | 2.57 (1.74, 3.31)* | 0.000* |
| Residence |  |  |  |  |  |
| Urban (reff.) | 250 | 11 | 1 | 1 |  |
| Rural | 6785 | 631 | 3.88 (1.74, 8.69)* | 3.27 (1.45, 7.38)* | 0.004* |
| Sex of house hold head |  |  |  |  |  |
| Female (reff.) | 4494 | 437 | 1 | 1 |  |
| Male | 2541 | 205 | 0.65 (0.54, 0.78)* | 0.67 (0.56, 0.81)* | 0.007* |
| ANC fellow up |  |  |  |  |  |
| No (reff.) | 4490 | 447 | 1 | 1 |  |
| Yes | 2545 | 195 | 0.84 (0.70, 1.01) | 0.89 (0.73, 1.08)* | 0.231 |
| Anemic Child |  |  |  |  |  |
| Yes (reff.) | 1452 | 153 | 1 | 1 |  |
| No | 5583 | 489 | 0.61 (0.50, 0.74)* | 0.67 (0.55, 0.83)* | 0.000* |
| Exclusively breastfeeding |  |  |  |  |  |
| Yes (reff.) | 4310 | 428 | 1 | 1 |  |
| No | 2725 | 214 | 1.52 (1.26, 1.83)* | 1.26 (1.02, 1.55)* | 0.030* |
| Mother's age |  |  |  |  |  |
| 34 and below | 3100 | 263 | 1 | 1 |  |
| 35–40 | 3459 | 321 | 1.29 (1.08, 1.55)* | 1.27 (1.06, 1.52)* | 0.010* |
| 41 and above | 476 | 58 | 2.0 (1.147, 2.74)* | 2.18 (1.59, 2.98)* | 0.000* |
| Child vaccinated (reff.= no) |  |  |  |  |  |
| No (reff.) | 2809 | 249 | 1 | 1 |  |
| Yes | 4226 | 393 | 0.58 (0.48, 0.70)* | 0.72 (0.59, 0.88)* | 0.002* |
| **NB**: *Significant at p-value < 0.05.  **Abbreviations:** reff.: reference  LRT = LR chi^2^(10) = 142.32 (p < 0.000) | | | | | |

**Discussion**

Although there have been many advancements and measures to increase newborn survival, U5CMR is still a significant public health issue in the SSA, with Ethiopia being severely afflicted. Therefore, to ascertain U5CMR in Ethiopia's pastoral regions, the authors carried out this retrospective follow-up investigation. Accordingly, 84 deaths per 1,000 live births were reported to represent the overall incidence of U5CMR in Ethiopia's pastoral regions. This result is in line with a prior investigation in SSA (81 deaths per 1,000 live births) [3], Ethiopia (61 deaths per 1,000 live births) [1], and Nigeria (104 deaths per 1,000 live births). The authors results, however, are lower than those of other investigations carried out in Ethiopia (273 deaths per 1,000 live births), Somalia (133 deaths per 1,000 live births), Chad (127 deaths per 1,000 live births), the Central African Republic (124 deaths per 1,000 live births), Sierra Leone (114 deaths per 1,000 live births), Mali (111 deaths per 1,000 live births) [3], and Ghana (250 deaths per 1,000 live births) [10].

On the other hand, this results are significantly greater than the U5CMR incidence reported in northern Ethiopia (35.62 deaths per 1,000 live births) [5], the Somali region of Ethiopia (57 deaths per 1,000 live births) [12], the rural area of Eastern Ethiopia (28.37 deaths per 1,000 live births) [13], the Tigray region of northern Ethiopia (62.5 deaths per 1,000 live births) [14], and Ethiopia (36.7 deaths per 1,000 live births) [15]. The foregoing variables in study designs, sample sizes, study periods, study sites, and sociodemographic characteristics of study participants may help to explain the variances between studies. According to this study, children under the age of five who were not anemic had a 33% lower risk of dying than those who were. A study conducted in Ethiopia, Jimma University Specialized Hospital, Gedeo Zone, and Sekota Waghemra Zone of northern Ethiopia also demonstrated that severe anemia had a decreased case fatality rate [9, 16–18]. Similarly, research from South Africa and Niger [19, 20] confirmed that exposed children have a greater risk of dying before the age of five. This is due to the fact that anemia will result in less compliance overall as well as an increase in infection incidence and heart failure risk. The other rationale for this study's findings is that anemia in young children might result in growth retardation and preterm birth, which are the biggest risk factors for mortality among under-five-year-olds.

The hazards of time-to-death for under-five children were lower among male household heads than female household heads for the predictor variable, sex of household heads. Children under the age of five who came from moms with fewer overall children were at the lowest risk of dying [21–23].

Additionally, among children under the age of five, exclusively breastfeeding was a significant predictor of time to death. This research showed that, compared to their peers who exclusively breastfed, children who did not start breastfeeding were at a higher risk of dying. This discovery is in line with a study carried out in Ethiopia [1]. According to research done in other SSA nations, breastfeeding can reduce the risk of time-to-death in children under the age of five by 16% if it begins within the first day of delivery and by as much as 22% if it begins within an hour [24].

The time to death among children under the age of five was strongly correlated with the mother's occupation. As a result, babies born to moms who work in agriculture have a higher risk of dying than babies born to mothers who do not work. This result conflicts with those of an Ethiopian study, which showed that the risks of under-five mortality were lower among children born to mothers who did not work than among mothers who were employed in the agricultural sector [21]. Because moms in the agricultural sector have higher incomes than mothers who aren't working, this discrepancy in results may be explained by differences in the socioeconomic and demographic status of the mothers. In the current study, the important risk factors for under-five-year-old children's time to death were shown to grow as mother's age increased. This result is in line with those of earlier research carried out in Sudan [25] and Suriname [26]. This may be related to the fact that older moms are more likely to experience pregnancy-related hazards, such as poor neonatal outcomes, according to research [27]. This study, however, contrasted with one conducted in Nigeria [28]. Interventions must therefore be coordinated in order to decrease dangerous conditions like pregnancy among older mothers. Another key socio-demographic factor that affects the time-to-death among under-five children is place of residence. Compared to urban people, the children of rural residents were more likely to die before the age of five. This result is in line with research done in Ethiopia [29–31]. Finally, this study demonstrated that children who were not immunized had a higher risk of dying sooner than those who were. This result is in line with research done in Ethiopia [11].

**Strength and limitation of the study**

A large sample size, nationally representative data collection served as the foundation for the current investigation. Additionally, it was done using the most appropriate statistical Cox PH regression model analysis (survival model technique) to account for regional or national variations in the deaths of children under the age of five. Moreover, because the study is based on national survey data collection, it may help policymakers and program designers develop effective intervention strategies at both the national and regional levels. The EDHS is largely based on respondents' self-reports, which raises the possibility of recall bias given the interview's retrospective study design and the high number of missing values in the 2016 EDHS data. Moreover, some significant predictor variables, such as the gestational mother's age, were left out of the study. The investigators should be advised that when their data structure is hierarchical, as on the 2016 EDHS data set, robust parametric models are preferable to conventional Cox PH regression methods, and further study using a prospective follow-up study design would better compensate for these limitations. Moreover, this investigation was done 07 years back so it is unlikely to reflect the latest status of mortality rate in the pastoral region of Ethiopia.

**Conclusions**

In this study, the authors found a higher rate of under-five deaths than the national figure. A child vaccinated, exclusively breastfeeding, mother’s occupation, sex of household head, anemic Child, mother's age, and residence were found to be the most influential predictors for time-to-death. Therefore, to lower the high incidence of under-five mortality, the government should focus on the pastoral regional states of Ethiopia.

**Abbreviations**

AHR: **A**djusted **H**azard **R**atio; ANC: **A**nte-**N**atal **C**are; CHR: **C**rude **H**azard **R**atio; CI: **C**onfidence **I**nterval; CSA: **C**entral **S**tatistical **A**gency; EDHS: **E**thiopia **D**emographic **H**ealth **S**urvey; KM: **K**aplan-**M**eier; LRT: **L**ikelihood **R**atio **T**est; MDG4: **F**ourth **M**illennium **D**evelopment **G**oal; MOH: **M**inister of **H**ealth; PH: **P**roportional-**H**azards; SSA: **S**ub-**S**aharan **A**frican; U5CMR: **U**nder-**5** **C**hildren **M**ortality **R**ate

**Acknowledgments**

We greatly acknowledge MEASURE DHS for permitting access to the 2016 EDHS data set.

Moreover, both authors wish to thanks a lot the anonymous reviewers and editor for their several insightful and constructive comments.

**Authors’ contributions**

BTH conceived the study. GGG and BTH analyzed the data, drafted the manuscript, and extensively reviewed the article. All authors have read and approved the final manuscript.

**Funding**

No funding was obtained for this study.

**Availability of data and materials**

The data set is available online and anyone can access it from [www.measuredhs.com](http://www.measuredhs.com).

**Consent for publication**

Not applicable.

**Competing interests**

The authors declare that they have no conflict of interest.

**Reference**

1. Zewudie AT, Gelagay AA, Enyew EF. Determinants of under-five child mortality in Ethiopia: analysis using Ethiopian demographic health survey, 2016. Int J Pediatr. 2020; 2020. <https://doi.org/10.1155/2020/7471545>.
2. Gebretsadik S, Gabreyohannes E. Determinants of under-five mortality in high mortality regions of Ethiopia: an analysis of the 2011 Ethiopia demographic and health survey data. Int J Popul Res. 2016. <http://dx.doi.org/10.1155/2016/1602761>.
3. Woldeamanuel BT. Socioeconomic, Demographic, and Environmental Determinants of Under-5 Mortality in Ethiopia: Evidence from Ethiopian Demographic and Health Survey, 2016. Child Development Research. 2019.<https://doi.org/10.1155/2019/1073782>.
4. L. Alkema, D. Chou, D. Hogan et al., “Global, regional, and national levels and trends in maternal mortality between 1990 and 2015, with scenario-based projections to 2030: a systematic analysis by the Maternal Mortality Estimation Inter-Agency Group,” *The Lancet*, vol. 387, no. 10017, pp. 462–474, 2016. <https://doi.org/10.1016/S0140-6736(15)00838-7>
5. Abraha HE, Belachew AB, Ebrahim MM, Tequare MH, Adhana MT, Assefa NE. Magnitude, trend, and causes of under-five mortality from Kilite-Awlaelo health demographic surveillance database, northern Ethiopia, 2009–2017. BMC Public Health (2020) 20:1465, <https://doi.org/10.1186/s12889-020-09554-z>.
6. Y G, S B. Survival analysis of under-five mortality of children and its associated risk factors in Ethiopia. J Biosens Bioelectron. 2016; 7 (3). <https://doi.org/10.4172/2155-6210.1000213>.
7. Bryce J, Victora C, Berman P, Lawn J, Mason E, Starrs A. Fulfilling the Health Agenda for Women and Children. 2014.
8. Central Statistical Agency (CSA) [Ethiopia] and ICF. Ethiopia demographic and health survey 2016. Addis Ababa, Ethiopia, and Rockville: CSA and ICF; 2016.
9. Berhie KA, Gebresilassie HG. Logistic regression analysis on the determinants of stillbirth in Ethiopia. Maternal Health, Neonatology, and Perinatology (2016) 2:10. <https://doi.org/10.1186/s40748-016-0038-5>.
10. Acheampong GK, Avorgbedor YE. Determinants of under Five Mortality in Ghana; A Logistic Regression Analysis Using Evidence from the Demographic and Health Survey (1988- 2014). *American Journal of Public Health Research, 2017, Vol. 5, No. 3, 70-78.* Available online at: <http://pubs.sciepub.com/ajphr/5/3/4>.
11. Fenta SM, Fenta HM (2020) Risk factors of child mortality in Ethiopia: Application of multilevel two-part model. PLoS ONE 15(8):e0237640. <https://doi.org/10.1371/journal.pone.0237640>.
12. Farah AE, Abbas AH, Ahmed AT. Trends of admission and predictors of neonatal mortality: a hospital based retrospective cohort study in Somali region of Ethiopia. PLoS One. 2018; 13:9.
13. Desta BN, et al. Neonatal mortality and its risk factors in eastern Ethiopia: a prospective cohort study in Kersa health and demographic surveillance system (Kersa HDSS). Epidemiol Biostat Public Health. 2016; 13:4.
14. Mengesha HG, et al. Survival of neonates and predictors of their mortality in Tigray region, NorthernEthiopia. BMC Pregnancy Childbirth. 2014; 16(1):202.
15. Mekonnen Y, et al. Neonatal mortality in Ethiopia: trends and determinants. BMC Public Health. 2013; 13:483.
16. Jarso H, Workicho A, Alemseged F. Survival status and predictors of mortality in severely malnourished children admitted to Jimma University Specialized Hospital from 2010 to 2012, Jimma, Ethiopia: a retrospective longitudinal study. *BMC Pediatr*. 2015; 15:76.
17. Girum T, Kote M, Tariku B, Bekele H. Survival status and predictors of mortality among severely acute malnourished children <5 years of age admitted to stabilization centers in Gedeo Zone: a retrospective cohort study. Therapeutics and Clinical Risk Management 2017:13.
18. Desta KS. Survival status and predictors of mortality among children aged 0–59 months with severe acute malnutrition admitted to stabiliza­tion center at Sekota Hospital Waghemra Zone. *J Nutr Disord Ther*. 2015; 5:160.
19. De Maayer T, Saloojee H. Clinical outcomes of severe malnutrition in a high tuberculosis and HIV setting. *Arch Dis Child*. 2011; 96(6):560–564.
20. Lapidus N, Minetti A, Djibo A, et al. Mortality risk among children admitted in a large-scale nutritional program in Niger, 2006. *PLoS One*. 2009; 4(1):e4313.
21. Woldeamanuel BT and Gelebo KK. Statistical analysis of socioeconomic and demographic correlates of perinatal mortality in Tigray region, Ethiopia: a cross sectional study. BMC Public Health (2019) 19:1301. <https://doi.org/10.1186/s12889-019-7642-z>.
22. Mbiba V. Factors associated with perinatal mortality in Umguza and Bubi rural areas, 2015- the effect of maternal human immunodeficiency virus status. UNSPECIFIED: UNSPECIFIED thesis; 2015.
23. Saffron, Lale Say, João-Paulo Souza, Carol J Hogue, Dinorah L Calles, A Metin Gülmezoglu and Rosalind Raine: The relationship between maternal education and mortality among women giving birth in health care institutions: Analysis of the cross sectional WHO Global Survey on Maternal and Perinatal Health; BMC Public Health2011 11:606, <https://doi.org/10.1186/1471-2458-11-60>.
24. Edmond KM, Kirkwood BR, Amenga-Etego S, Owusu-Agyei S, Hurt LS. Effect of early infant feeding practices on infection-specific neonatal mortality: an investigation of the causal links with observational data from rural Ghana. Am J Clin Nutr. 2007;86(4):1126–31.
25. Bashir AO, et al. Neonatal mortality in Sudan. BMC Public Health. 2013; 13:287.
26. Zijlmans W, Hindori-Mohangoo A. Determinants of neonatal mortality in Suriname: preliminary findings from a perinatal and infant mortality survey. Ann Glob Health. 2015; 81(1):121–2.
27. Mutz-Dehbalaie, et al. Perinatal mortality and advanced maternal age. Gynecol Obstet Investig. 2014; 77(1):50–7.
28. Yaya S, Ekholuenetale M, Tudeme G, Vaibhav S, Bishwajit G, Kadio B. Prevalence and determinants of childhood mortality in Nigeria. BMC Public Health (2017) 17:485.DOI 10.1186/s12889-017-4420-7.
29. S. Yaya,G. Bishwajit, F.Okonofua, O. A.Uthman, and J. P. van Wouwe, “Under fivemortality patterns and associatedmaternal risk factors in sub-Saharan Africa: A multi-country analysis,” *PLoS ONE*, vol. 13, no. 10, article no. e0205977, 2018.
30. N. S. Mugo, K. E. Agho, A. B. Zwi, E. Y. Damundu, and M. J. Dibley, “Determinants of neonatal, infant and under-five mortality in a war-affected country: analysis of the 2010 Household Health Survey in South Sudan,” *BMJ Global Health*, vol. 3, no. 1, article no. e000510, 2018.
31. Berelie Y, Yismaw L, Tesfa E, Alene M.Risk factors for under-five mortality in Ethiopia: Evidence from the 2016 Ethiopian Demographic and Health Survey.*S Afr J Child Health* 2019;13(3):137-140. <https://doi.org/10.7196/SAJCH.2019.v13i3.1645>.

| **Additional file** | |  |  |
| --- | --- | --- | --- |
|  |  |  |  |
| **Additional file 1: Table S1.** Survival experience comparison | | | |
| **Ns** | **Variables** | **chi^2^(df)** | **LRT p-value** |
| 1 | Type of birth | 0.07 | 0.798 |
| 2 | Father's education level | 3.1 | 0.213 |
| 3 | Mother’s occupation | 49.09 | 0.000* |
| 4 | Region | 0.52 | 0.771 |
| 5 | Residence | 12.92 | 0.0003* |
| 6 | Toilet facilities | 0.67 | 0.412 |
| 7 | Mother’s education level | 0.13 | 0.717 |
| 8 | Sex of household head | 22.47 | 0.000* |
| 9 | Sex of child | 0.68 | 0.410 |
| 10 | ANC fellow up | 3.42 | 0.064 |
| 11 | Modern contraceptive used | 0.001 | 0.990 |
| 12 | Anemic Child | 25.08 | 0.000* |
| 13 | Exclusive breastfeeding | 20.36 | 0.000* |
| 14 | Mother's age | 22.25 | 0.000* |
| 15 | Child vaccinated | 37.47 | 0.000* |
| **LRT: Log-Rank Test.** | | | |

**Figures with its caption**

Figure 2: Survival curve by Mothers age of children

Figure 3: Survival curve by residence place of children

Figure 4: Survival curve by sex of household head

Figure 5: Survival curve by anemic level of mother’s

Figure 6: Survival curve child vacination

Figure 7: Survival curve by breastfeeding of child

Figure 6: Survival curve by child vaccination status

**Appendix**

STATA-codes for data Analysis

** This used to test Cox proportional assumption**

stset survival_time, failure(status==1) scale(1)

estat phtest, detail

** After the PH assumption had been checked, Analysis Bi-variable Cox PH model **

stset survival_time, failure(status==1) scale(1)

stcox i.Type_of_birth i.Fathers_educational_level i.Mothers_occupation i.Region i.Residence i.Toilet_facilities i.Mothers_educational_level i.Sex_of_household_head i.Sex_of_child i.ANC_fellow_up i.Modern_contraceptive_used i.Anemic_level_of_mothers i.Breastfeeding i.Mothers_age i.Child_vaccination_status

** After the Bi-variable Cox PH model analysis fit p-value <0.25 in Multivariable analysis **

**This used to model Multivariable Cox PH analysis**

stset survival_time, failure(status==1) scale(1)

stcox i.Mothers_occupation i.Residence i.Sex_of_household_head i.ANC_fellow_up i.Anemic_level_of_mothers i.Breastfeeding i.Mothers_age i.Child_vaccination_status

**This used for figures of KM-curves of category predictors**

stset survival_time, failure(status)

sts graph, by(i.Mothers_age) ytitle(Survival probability of under-five children) xtitle(Survival time of under-five children) title(KM-curve for Mother’s age differentials) subtitle(Fig 2: Survival curve by Mother’s age) legend(on)

stset survival_time, failure(status)

sts graph, by(i.Residence) ytitle(Survival probability of under-five children) xtitle(Survival time of under-five children) title(KM-curve for Residence place differentials) subtitle(Fig 2: Survival curve by residence place) legend(on)

stset survival_time, failure(status)

sts graph, by(i.Sex_of_household_head) ytitle(Survival probability of under-five children) xtitle(Survival time of under-five children) title(KM-curve for sex of household head differentials) subtitle(Fig 3: Survival curve by sex of household head) legend(on)

stset survival_time, failure(status)

sts graph, by(i.Anemic_level_of_mothers) ytitle(Survival probability of under-five children) xtitle(Survival time of under-five children) title(KM-curve for Anemic_level_of_mothers differentials) subtitle(Fig 4: Survival curve by Anemic_level_of_mothers) legend(on)

stset survival_time, failure(status)

sts graph, by(i.Child_vaccination_status) ytitle(Survival probability of under-five children) xtitle(Survival time of under-five children) title(KM-curve for Child vaccination status differentials) subtitle(Fig 5: Survival curve by Child vaccination status) legend(on)

stset survival_time, failure(status)

sts graph, by(i.Breastfeeding) ytitle(Survival probability of under-five children) xtitle(Survival time of under-five children) title(KM-curve for child breastfeeding differentials) subtitle(Fig 6: Survival curve by child breastfeeding) legend(on)

*The log-rank test was used to test whether there is significant d/ce b/n predictors.*

stset survival_time, failure(status==1) scale(1)

sts test Type_of_birth, logrank

sts test Fathers_educational_level, logrank

sts test Mothers_occupation, logrank

sts test Region, logrank

sts test Residence, logrank

sts test Toilet_facilities, logrank

sts test Mothers_educational_level, logrank

sts test Sex_of_household_head, logrank

sts test Sex_of_child, logrank

sts test ANC_fellow_up, logrank

sts test Modern_contraceptive_used, logrank

sts test Anemic_level_of_mothers, logrank

sts test Breastfeeding, logrank

sts test Mothers_age, logrank

sts test Child_vaccination_status, logrank
