# Supplementary material for: Predictors of time to death among under-five children in pastoral regions of Ethiopia: A retrospective follow-up study
Source: PLoS One. 2024 Jul 31;19(7):e0304662. doi: 10.1371/journal.pone.0304662 (PMC11290679; doi:10.1371/journal.pone.0304662)
Supplement: Attachment — Submitted filename: Response to Reviewers_5.docx [file pone.0304662.s011.docx]

**Author’s response to reviews**

**Title:** Predictors of time to death among under-five children in pastoral regions of Ethiopia: a retrospective follow-up study

**Authors:**

Bsrat Tesfay Hagos ([bsrattesfaygu@gmail.com](mailto:bsrattesfaygu@gmail.com)) | **ORCID**: [https://orcid.org/0009-0001-0487-7577](https://orcid.org/0009-0001-0487-7577?lang=en)

Gebru Gebremeskel Gebrerufael ([gebrugebremeskel12@gmail.com](mailto:gebrugebremeskel12@gmail.com)) | **ORCID**: <https://orcid.org/0000-0003-2795-8529>

**Version:** 4 Date: 02-May-2024

**Author’s response to reviews:**

**Response to editors**

Dear editors-in-chief,

The authors have been enclosed a revised version of the manuscript in **PLOS ONE** research article format, entitled “Predictors of time to death among under-five children in pastoral regions of Ethiopia: a retrospective follow-up study**"** As detailed in the Response to Editors Comments on the following pages, the authors have attempted to address every one of the editors' comments in this revised version of the manuscript.

The authors feel that this revised version of the manuscript fits very well within the scope of **PLOS ONE** because it covers a number of computational approaches and issues that are important to the analysis of public health issues, as stated in my cover letter that went along with this original revised version of the manuscript submission.

With warm regards,

**Bsrat Tesfay Hagos**

On the behalf of the other authors

**Response to Editor and Reviewers comments:**

**Title:** Predictors of time to death among under-five children in pastoral regions of Ethiopia: a retrospective follow-up study

**Authors**: Gebru Gebremeskel Gebrerufael and Bsrat Tesfay Hagos

**MS ID**: PONE-D-23-08757R3

**Journal**: **PLOS ONE**

**Article type**: Research article

The authors would like to thank you so much to the anonymous editors and reviewers for their precious time and understanding review of this manuscript and for all of their supportive and constructive comments. They raise significant concerns, and their input is very supportive of improving the main manuscript. The authors agree with almost all their comments and have revised this main manuscript accordingly. The authors provided underneath a **point-by-point** response in **purple colored,** which describes attempts to solve all of the requested revisions in our main manuscript.

**Editor’s comments:**

**Comment 1:** Please remove the extra author included after the completion of the substantial review of the manuscript. 
Response 1: Thank you very much Reviewer. The authors have removed according your best suggestion.

**Comment 2:** Please review your reference list to ensure that it is complete and correct. If you have cited papers that have been retracted, please include the rationale for doing so in the manuscript text, or remove these references and replace them with relevant current references. Any changes to the reference list should be mentioned in the rebuttal letter that accompanies your revised manuscript. If you need to cite a retracted article, indicate the article’s retracted status in the References list and also include a citation and full reference for the retraction notice.

**Author Response 2:** Thank you so much for your constructive and impressive suggestions. Therefore, the authors have corrected according your suggestions overall the revised manuscript. See on the references section on page #18-22 and #line 323-419.
